# Identification of the NA^+^/K^+^-ATPase α-Isoforms in Six Species of Poison Dart Frogs and their Sensitivity to Cardiotonic Steroids

**DOI:** 10.1007/s10886-023-01404-7

**Published:** 2023-03-06

**Authors:** Katherine Medina-Ortiz, Felipe Navia, Claudia Mosquera-Gil, Adalberto Sánchez, Gonzalo Sterling, Leonardo Fierro, Santiago Castaño

**Affiliations:** Laboratorio de Herpetología Y Toxinología, Department of Physiological Sciences, Faculty of Health, Universidad del Valle, Cali, Colombia

**Keywords:** Frogs, Toxins, Resistance, Mutations, Membrane protein, Transporters, RNA-seq, Homology

## Abstract

Cardiotonic steroids (CTS) are a group of compounds known to be toxic due to their ability to inhibit the Na^+^/K^+^-ATPase (NKA), which is essential to maintain the balance of ions in animal cells. An evolutionary strategy of molecular adaptation to avoid self-intoxication acquired by CTS defended organisms and their predators is the structural modification of their NKA where specific amino acid substitutions confer resistant phenotypes. Several lineages of poison dart frogs (Dendrobatidae) are well known to sequester a wide variety of lipophilic alkaloids from their arthropod diet, however there is no evidence of CTS-sequestration or dietary exposure. Interestingly this study identified the presence of α-NKA isoforms (α_1_ and α_2_) with amino acid substitutions indicative of CTS-resistant phenotypes in skeletal muscle transcriptomes obtained from six species of dendrobatids: *Phyllobates aurotaenia*, *Oophaga anchicayensis*, *Epipedobates boulengeri*, *Andinobates bombetes*, *Andinobates minutus,* and *Leucostethus brachistriatus,* collected in the Valle del Cauca (Colombia). *P. aurotaenia*, *A. minutus*, and *E. boulengeri* presented two variants for α_1_-NKA, with one of them having these substitutions. In contrast, *O. anchicayensis* and *A. bombetes* have only one α_1_-NKA isoform with an amino acid sequence indicative of CTS susceptibility and an α_2_-NKA with one substitution that could confer a reduced affinity for CTS. The α_1_ and α_2_ isoforms of *L. brachistriatus* do not contain substitutions imparting CTS resistance. Our findings indicate that poison dart frogs express α-NKA isoforms with different affinities for CTS and the pattern of this expression might be influenced by factors related to evolutionary, physiological, ecological, and geographical burdens.

**Supplementary Information:**

The online version contains supplementary material available at 10.1007/s10886-023-01404-7.

## Introduction

Cardiotonic steroids (CTS) are a type of toxins predominantly found in plants, but also in species of insects and vertebrates (Agrawal et al. 2012; Mohammadi et al. 2022b). CTS are characterized by the inhibition of the Na^+^/K^+^-ATPase (NKA) transporter and are used in defense against predators by many species (Krenn and Kopp 1998; Steyn and van Heerden 1998). CTS are more abundantly found in plants (12 families reported, with the family *Apocynaceae* having the largest number of CTS-producing genera), insects (Families: Danaidae, Arctiidae, Chrysomelidae, Cerambycidiae, Aphididae, Pyrgomorphidae, and Lygaeidae), anurans of the family Bufonidae (true toads), and colubrid snakes of the genus *Rhabdophis*, which sequester the toxins from its bufophagic diet (Agrawal et al. 2012; Krenn and Kopp 1998; Mohammadi et al. 2022b; Rodriguez et al. 2017; Yoshida et al. 2020). Some of these animals produce CTS *de novo* and others are capable of tolerating and obtaining them from their host plant or pray and using them for their defense via sequestration (Mohammadi et al. 2022b). These species could be protected from toxic effects by selective insensitivity to CTS at their NKA binding site (Arbuckle et al. 2017). There is also evidence that mammals can produce endogenous cardiotonic steroids (ECTS) (Orlov et al. 2021), and that these ECTSs are able to regulate different cellular activities through the modulation of the NKA activity.

Research on target-site insensitivity in α-NKA isoforms as a means of resistance to CTS in anurans has predominantly focused on bufonid toads and some of their predators within Leptodactylidae. In a previous study, we characterized the presence of different isoforms for the α-NKA subunit in the skeletal muscle of the bufonid toad *Rhinella marina*, an anuran that produces CTS. The presence of transcripts predominantly encoding α_1_-NKA isoform and low levels of α_2_ and α_3_ isoforms were detected by RNA-seq (Medina-Ortiz et al. 2021). This study found substitutions like those reported in CTS-feeding insects and animals that eat toads in α_2_-NKA (Q111T) and α_3_-NKA (Q111L and G120R) isoforms, respectively, suggesting lower CTS affinity (Medina-Ortiz et al. 2021). The molecular docking results between these isoforms and different CTS showed that all three have a low affinity for CTS compared to the susceptible α_1_ isoform from *Sus scrofa* (pig). With this approach, the α_1_ isoform from *R. marina* showed the lowest CTS affinity (Medina-Ortiz et al. 2021).

Another study performed on Neotropical grass frogs of the genus *Leptodactylus*, which are known to feed on toads that are CTS producers (Chen and Chen 1933; Crossland and Azevedo-Ramos 1999), found two copies of the ATP1A1 gene (which codes for the α_1_ isoform) and only one copy of the ATP1A2 and ATP1A3 genes (which code for the α_2_ and α_3_ isoforms) (Mohammadi et al. 2021). ATP1A2 and ATP1A3, along with one copy of the ATP1A1 gene, lack any known substitutions that would confer CTS resistance. On the other hand, the other ATP1A1 paralog has substitutions that have been shown to confer CTS resistance (Mohammadi et al. 2021).

An extensive survey of genes and transcripts encoding α-NKA paralogs done by Mohammadi et al*.* (Mohammadi et al. 2022a; Mohammadi et al. 2021) included some species of dendrobatids within their datasets. Mohammadi et al*.* (Mohammadi et al. 2021) reported α-NKA isoforms in *Dendrobates auratus* that do not possess substitutions conferring CTS resistance. In contrast, Mohammadi et al. (2022a) reported duplicated sequences for α_1_-NKA found in *Oophaga pumilio,* one of which contains CTS-resistance conferring substitutions. More recently, Hernandez-Poveda et al*.* (Hernández Poveda 2022) showed that ATP1A1 was tandemly duplicated in *O. pumilio* and *Ranitomeya imitator* and has evolved independently from *Leptodactylus.* Currently, only the α-NKA isoforms of these three species of dendrobatids have been annotated and characterized. In this study, we analyzed the α-NKA isoforms found in *de novo* transcriptome assemblies from six dendrobatid species, *Phyllobates aurotaenia*, *Oophaga anchicayensis*, *Epipedobates boulengeri*, *Andinobates bombetes*, *Andinobates minutus,* and *Leucostethus brachistriatus.* The analysis of six species of dendrobatids belonging to different genera allowed us to trace the origin and the evolutionary history of CTS resistant phenotypes and provides the basis for further explorations on the chemical ecology of these species.

Most of the research on the detection of chemical compounds from skin extracts in dendrobatids has been toward the search for alkaloid-type toxins, leaving aside compounds of a different nature. Thus, there is not clear evidence of the presence of ECTS or the sequestration of CTS in Dendrobatidae (Daly et al. 1987, 2005; Santos et al*.* 2016). However, it cannot be ruled out that dendrobatids possess ECTS, nor that they can be exposed to CTS through their diet. This, considering the great diversity and distribution of plants of the family Apocynaceae (~ 74 genera, 395 species present in Colombia), and other families of plants in which CTS and steroidal alkaloids have been detected at different levels in tropical zones (Liliaceae, Ranunculacea, Moraceae, Leguminosae, Scrophulariaceae, Cruciferae, Sterculiaceae, Euphorbiaceae, Tiliaceae, and Celastraceae) (Agrawal et al. 2012; Bernal et al. 2019; Morsy 2017; Rangel-Churio 2015). Therefore, it is possible that some dendrobatids, which are sympatric with these plants, could have a diet that includes arthropod sources of CTS. Intriguingly, although it is unclear the synthetic origin of many of the alkaloid-type toxins found in dendrobatids, steroid alkaloids such as batrachotoxin (BTX), found in in the genus *Phyllobates*, share structural similarities with CTS (Heasley 2012); this could indicate a possible common biosynthetic route or precursors. BTX and CTS, both have a lipophilic steroid core, however the different groups attached to that steroid core, such as two pyrroles in BTX and one lactone group in CTS, cause their different physiological targets and biological activities (Heasley 2012).

Considering the above, this study identified two different α-NKA isoforms; α_1_ and α_2_-NKA in skeletal muscle transcriptomes from dendrobatid species. *Leucostethus brachistriatus*, *O. anchicayensis*, and *A. bombetes* were characterized as presenting a single variant for each of the two isoforms. In contrast, two potentially coding variants for the α_1_-NKA isoform and one single variant for the α_2_-NKA isoform were found in *P. aurotaenia*, *E. boulengeri*, and *A. minutus*. One of these α_1_-NKA variants shows molecular substitutions that suggest CTS resistance. Molecular docking analysis of the isoforms and different CTS showed that they have lower affinity for CTS than the susceptible α_1_-NKA isoform from *S. scrofa*.

## Material and Methods 

### Sampling and Tissue Processing

Tissue samples obtained from the sartorius skeletal muscle (long muscle covering the ventral surface of the thigh) were dissected from 6 dendrobatid species; *P. aurotaenia* (NCBI: txid152497), *O. anchicayensis* (NCBI: txid51949; separated from *Oophaga histrionica* by Posso-Terranova and Andrés in 2018), *E. boulengeri* (NCBI: txid92732), *A. bombetes (*NCBI: txid507665), *A. minutus* (NCBI: txid51959)*,* and *L. brachistriatus* (NCBI:txid2036793; In the past identified as *Colostethus fraterdanieli* by Grant and Castro [1998]; reassigned as *Colostethus brachistriatus* by Grant et al. [2017]; and transferred to *Leucostethus* by Marin et al. [2018]). All the individuals were collected in different regions of Valle del Cauca, Colombia (Table 1).

**Table 1:** Collection information of dendrobatid species collected in the department of Valle del Cauca, Colombia

*Species*	*Location*	*Coordinates* *Latitude/Longitude*	*Elevation* *(masl)*	*n*
*P. aurotaenia*	Bahía Málaga, Buenaventura, Chocó Biogeographical Region*, Valle del Cauca, Colombia	3.9813889 -77.3366944	0–50	3
*E. boulengeri*	Bahía Málaga, Buenaventura, Chocó Biogeographical Region*, Valle del Cauca, Colombia	3.989306019 -77.33152809	0–50	3
*A. minutus*	Bahía Málaga, Buenaventura, Chocó Biogeographical Region*, Valle del Cauca, Colombia	3.989306019 -77.33152809	0–50	3
*O. anchicayensis*	El Naranjo, Dagua, Colombia	3.783999744 -76.72099993	828	3
*A. bombetes*	Reserva Forestal de Yotoco, Yotoco, Colombia	3.875055981 -76.43594399	1604	2
*L. brachistriatus*	Universidad del Valle, Campus Meléndez, Cali, Colombia	3.373440044 -76.53460404	972	2

*The Chocó Biogeographical Region is also known as the Tumbes-Chocó-Magdalena region (Pérez-Escobar et al. 2019)

The sampled tissues were cut into small pieces, quickly transferred to 10 volumes of RNAlater (Life Technologies, USA), and stored at 4 °C for 48 h. Subsequently, the RNAlater was drained, and the tissues were stored at -80 °C (Huang et al. 2017). Later, the tissues were pulverized with a porcelain mortar under liquid nitrogen and the total RNA was purified using the RNeasy Plus Mini Kit (Qiagen, Germany) following the manufacturer's protocol, including the optional DNase digestion step. After extraction, the RNA was spectrophotometrically quantified by Nanodrop (Thermo Scientific Inc., USA) and the integrity of the RNA was verified electrophoretically.

### Ethics Statement

Animal use, procedures, and protocols were approved by the Institutional Committee of Care and Use of Laboratory Animals from Universidad del Valle**.** The permit for the collection of wild specimens for non-commercial scientific research purposes was granted to Universidad del Valle by the National Authority for Environmental Licenses (known as ANLA in Colombia) through Resolution 1070 of August 28, 2015.

The Colombian Ministry of home affairs certified (ratification No. 175 of February 26, 2015) that there is no presence of minoritarian communities in the areas of field collections. The Colombian Ministry of Environment and Sustainable Development granted this project with the Contract for Access to Genetic Resources and Derivative Products No. 143 (through Resolution 1348 of 2014).

### Sequencing and Bioinformatics

Total RNA from skeletal muscle was sent to Omega Bioservices for RNA sequencing (RNA-seq) of paired-reads libraries (100 bp; 2X; 20–25 million reads per sample). Prior to sequencing, RNA concentration and integrity was determined using a Nanodrop 2000c spectrophotometer (Thermo Scientific Inc.) and Agilent 2200 TapeStation system (Agilent Technologies, Santa Clara, CA, USA). PolyA mRNA from an input of 500 ng high quality total RNA (RINe > 8) was purified and fragmented. RNA sequencing libraries were prepared using TruSeq Stranded mRNA Library Prep Kit (Illumina, Inc., San Diego, CA, USA). The libraries were normalized, multiplex, pooled in two lanes, and subjected to cluster and pair read sequencing performed for 100 cycles on a HiSeq 2500 instrument (Illumina, Inc. San Diego, CA, USA), and the final data was stored on the Illumina BaseSpace platform. The demultiplexing and quality control of the reads (Phred quality scores ≥ 30) was conducted by Omega Bioservices.

The reads were assembled *de novo* with Trinity software (v 2.11.0 and 2.15.0; minContigLen = 200) (Grabherr et al. 2011; Haas et al. 2013). The NCBI BLASTx server (e-value < 10^–5^) was used to search transcripts coding for the α isoforms of NKA (http://blast.ncbi.nlm.nih.gov). The best BLAST results in terms of e-value were selected. For each transcript, open reading frames were predicted using ORFfinder (https://www.ncbi.nlm.nih.gov/orffinder/). The following criteria were considered for the selection of potential transcripts: a. The quality of the transcriptome assembly was examined using Bowtie2 and BUSCO algorithms (Langmead and Salzberg 2012; Manni et al. 2021); b. The length of the CDS (coding DNA sequence), CDS encoding amino acid sequences homologous to the α isoform of NKA with lengths greater than 700 residues (≥ 2000 bp) were considered; c. Best BLASTx results with e-values ​​ < 10^–5^ using as reference NKA proteins found in UniProt/Swiss-prot and GenBank databases; d. Visual analysis of predicted amino acid sequences to check the conservation of motifs isoform-specific (Duran et al. 2004; Sottejeau et al. 2010). Reads were realigned to the transcriptome assembly using Bowtie2 to check the coverage of reads per transcripts. Relative abundances and normalization were estimated by using Salmon (Patro et al. 2017). Salmon uses quasi-mapping approach to quantify the reads mapping to each transcript and provides measurements of transcript abundances as raw counts and transcripts per million (TPM) (Wagner et al. 2012; Zhang et al. 2017). TPM is a commonly used normalization method and provides a general overview of gene expression level of each transcript within of each sequenced library.

The amino acid sequences, translated from the ORF, were aligned using the MUSCLE algorithm and annotated to search for potential candidate sites and substitutions that could potentially cause a reduction in the affinity between the toxins of interest and NKA. In the alignments, the residue numbering of the porcine α_1_ sequence after post-translational cleavage of the first five amino acid residues, as is found in *S. scrofa* NKA crystals, was used for simplicity.

### Phylogenetic Analysis

The evolutionary relationships among all α-NKA isoform amino acid sequences were inferred by Maximum Likelihood Estimations (MLE) and JTT (Jones-Taylor-Thornton) matrix-based model in MEGA11 (Tamura et al. 2021). To estimate the best substitution model, the option Models of MEGA11 was used and the best model was selected based on the AICc criterion. The ML heuristic method was performed with the nearest-neighbor-interchange (NNI). The initial trees for the heuristic search were obtained automatically by applying the Neighbor-Joining and BioNJ algorithms to a matrix of pairwise distances estimated using the JTT model, and then selecting the topology with the highest log-likelihood value. A discrete Gamma distribution was used to model the differences in evolutionary rates between sites (5 categories (+ G, parameter = 0.2492)). For divergence time calibration, an indirect calibration was used derived from calibrated with divergence times obtained from www.timetree.org.

### Homology Modeling and Molecular Docking

A homology modeling approach was used to predict the three-dimensional structure of proteins from known amino acid sequences. Structural models of α-isoforms of the non-crystallized sequences were fit structurally and energetically (minimum energy) to crystallized models reported in PDB and co-crystallized with CTS toxins. The α-NKA subunit of *S. scrofa* (Access code: 7EVX, www.rcsb.org) was used as a template since it is co-crystallized with CTSs in a state of high affinity.

The amino acid sequences encoding α-NKA isoforms of each anuran species were reconstructed to their three-dimensional version by homology using the SWISS-MODEL server (http//swissmodel.expasy.org/) (Waterhouse et al. 2018). To estimate the binding energy (BE) of the ligand–protein complexes, different CTSs were docked with each isoform receptor using the AutoDock Vina software (Trott and Olson 2010). An area in the binding pocket for CTSs was defined using a three-dimensional grid (coordinates: -31.8156, 37.2106, 62.5142 and a size of 31.695, 30.4563, 29.7248 Å for the x, y, and z axes, respectively) to perform molecular docking interactions. An additional property called *spacing*, was used, which indicates the separation between the points within the three-dimensional box. The *S. scrofa* α_1-_NKA isoform was used as a control receptor to dock the same ligands; these complexes were chosen as references due to their susceptibility to CTSs (Gable et al. 2017). The proposed ligands (Table 2) were reconstructed using the canonical SMILES codes from PubChem NCBI (pubchem.ncbi.nlm.nih.gov).

**Table 2:** Cardenolides used as ligands in molecular docking analysis

Ligands		Canonical Code SMILES
*Ouabain*	OBN	CC1C(C(C(C(O1)OC2CC(C3(C4C(CCC3(C2)O)C5(CCC(C5(CC4O)C)C6 = CC(= O)OC6)O)CO)O)O)O)O
*Ouabagenin*	OBG	CC12CC(C3C(C1(CCC2C4 = CC(= O)OC4)O)CCC5(C3(C(CC(C5)O)O)CO)O)O
*Digoxigenin*	DIXG	CC12CCC(CC1CCC3C2CC(C4(C3(CCC4C5 = CC(= O)OC5)O)C)O)O
*Cymarin (or cymarine)*	CMR	CC1C(C(CC(O1)OC2CCC3(C4CCC5(C(CCC5(C4CCC3(C2)O)O)C6 = CC(= O)OC6)C)C = O)OC)O
*Strophanthins*	SPT	CC12CCC3C(C1(CCC2C4 = CC(= O)OC4)O)CCC5(C3(CCC(C5)O)C = O)O
*Oleandrin*	OLD	CC1C(C(CC(O1)OC2CCC3(C(C2)CCC4C3CCC5(C4(CC(C5C6 = CC(= O)OC6)OC(= O)C)O)C)C)OC)O

### Statistical Analysis

Principal component analysis (PCA) was performed considering the predicted top-five ranking poses with best scores (five lowest BE values) for each complex. This analysis allows trends in binding affinity of each CTS-NKA α complex to be visualized. In addition, the absolute values of the BE for the best pose estimated for each receptor were adjusted for statistical analyses using generalized linear models (GLM) with Gamma distribution and identity function. Subsequently, an ANOVA (deviation analysis in GLM) was performed to evaluate the statistical significance between receptors and ligands on the response variable BE. Differences between means were examined by Tukey’s post hoc test; p-values < 0.05 were considered statistically significant for all tests. These analyses were performed with R software (version 4.0.2, http://www.r-project.org).

## Results

### Identification of Transcripts Encoding α Isoforms in Dendrobatid Skeletal Muscle

**Fig. 1:**
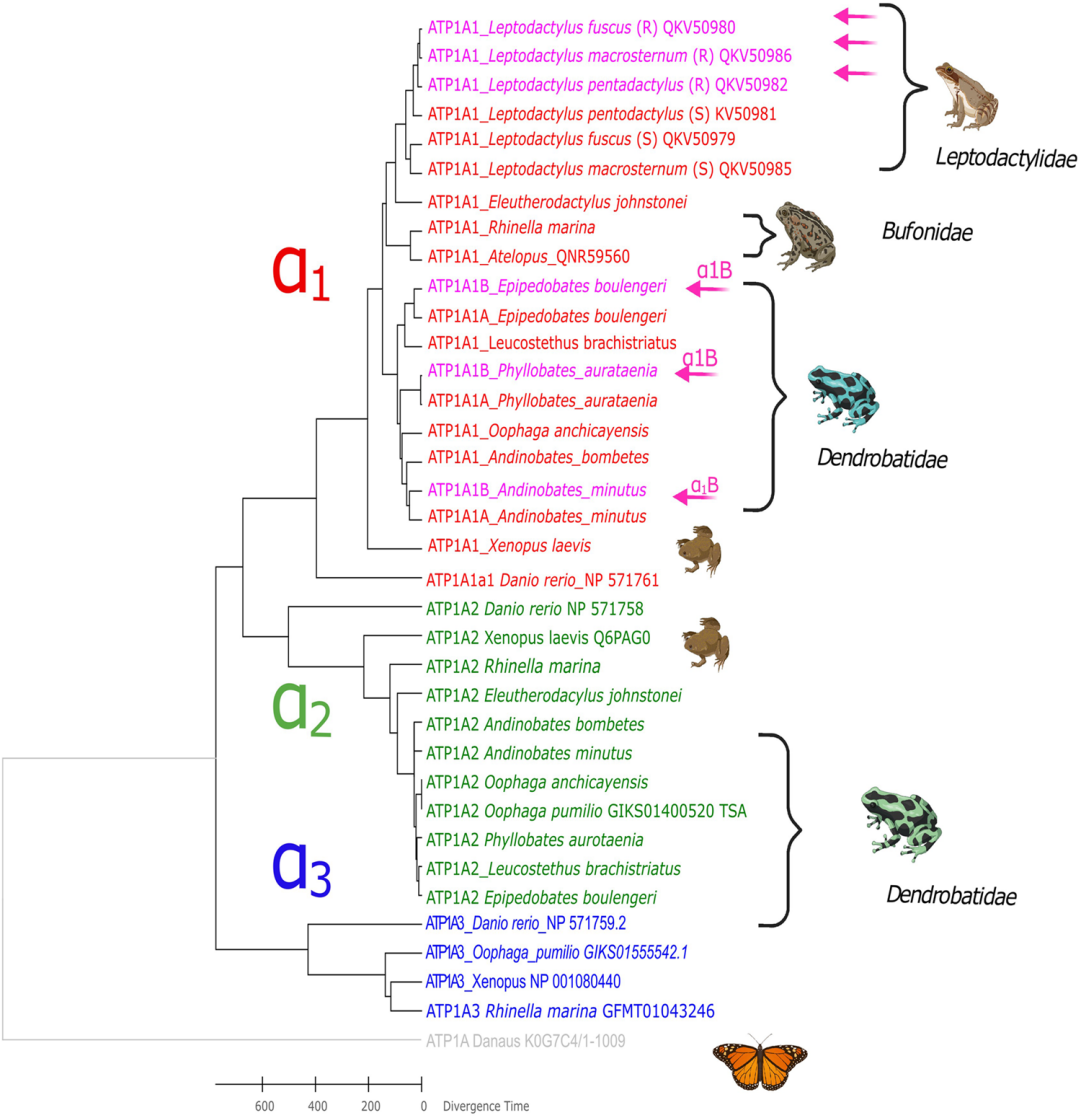
Maximum likelihood phylogeny inferred from amino acid sequences encoding α-NKA isoforms. Six species of the anuran families Dendrobatidae, Leptodactilidae, Pipidae, Electherodactylidae, and Bufonidae and one species of fish, zebrafish (*Danio rerio*), were included in the analysis (36 amino acid sequences; 1035 positions in the final dataset). The monarch butterfly, *Danaus plexippus*, was used as an outgroup of the α isoform paralogs. Each paralog is highlighted with different font colors; α_1_: red (α_1_B is highlighted in pink), α_2_: green, α_3_: blue. Pink arrows show α1 variants with RD substitutions previously associated with OBN resistance. Animal cartoons were taken from BioRender.com

Most tetrapod vertebrates studied to date have at least three α-NKA subunit isoforms encoded by different paralogous genes: α_1_, α_2_, and α_3_ (Saez et al. 2009). The alignments and phylogenetic analysis estimated based on the α-NKA from different lineages (mammals, anurans, and fish) show that the transcripts identified as α-NKA for the dendrobatid species are clustered with the α_1_ and α_2_ paralogs (Fig. 1).

All α_1_-NKA isoforms identified in this study had a length of 1022 residues, except for *L. brachistriatus*, whose α_1_ was characterized as having a length of 1024 amino acids (Table 3). All the α_2_-NKA isoforms identified in these transcriptomes had a length of 1020 amino acids. No transcripts encoding the α_3_-NKA isoform were identified in these transcriptomes.

**Table 3:** α-NKA isoforms identified in the transcriptomes from skeletal muscle of six Dendrobatidae species

Species	No. clean reads	Isoform	Contig length	CDS length	Amino acid length	Coverage^1^ (%)	Mean Depth^2^ (X)	TPM^3^	No. reads/Salmon
*P. aurotaenia*	24,775,019	α_1_A	3942	3069	1022	99.6	145.2	22.3	2875.4
		α_1_B	3949	3069	1022	99.5	174.8	56.2	7481.2
		α_2_	3818	3063	1020	96.9	321.2	59.1	7598.4
*E. boulengeri*	23,842,688	α1A	3425	3069	1022	100	208.68	52.5	4166.5
		α1B	3437	3069	1022	100	207.48	33.1	2625.5
		α_2_	5912	3063	1020	100	224.17	74.9	10,449.0
*A. minutus*	20,971,055	α1A	3529	3069	1022	99	246.4	35.7	4905.4
		α1B	3466	3069	1022	99.9	231.5	32.9	4516
		α_2_	3610	3063	1020	100	464.1	64.6	9134
*O. anchicayensis*	27,529,856	α_1_	3404	3069	1022	100	187	36.1	6318.4
		α_2_	3770	3063	1020	100	194.6	41	8002.2
*A. bombetes*	27,816,978	α_1_	3434	3069	1022	100	224	34.1	5535.6
		α_2_	3954	3063	1020	100	434.5	47.6	8974
*L. brachistriatus*	22,559,851	α_1_	3456	3075	1024	99	705	73.7	9667.8
		α_2_	4652	3063	1022	100	317.8	46.2	8373.6

1. Coverage (Samtools): Percentage of covered bases. This value refers to the percentage of bases of specific transcript that is covered with certain depth. Samtools (htslib.org)

2. Mean depth (Samtools): mean depth of coverage. This value is calculated as the number of bases of all short reads that match a specific transcript divided by its length

3. TPM: Transcripts per million

Based on the alignments, the transmembrane segments are highly conserved in both α_1_-NKA and α_2_-NKA paralogs and orthologs (Supplemental Dataset 1). The extracellular loop with the greatest variability between sequences with CTS-susceptible and CTS-resistant phenotypes was the loop between the first and second transmembrane segments (L1/2), followed by L7/8, whereas the loops L3/4 and L5/6 are highly conserved.

The species *L. brachistriatus*, *O. anchicayensis*, and *A. bombetes* were characterized as presenting a single variant for each paralog. In contrast, two potentially coding variants for the α_1_-NKA isoform and one single variant for α_2_-NKA isoform were found in skeletal muscle transcriptomes from *P. aurotaenia*, *E. boulengeri*, and *A. minutus*. The main differences between both variants occurred in L1/2. The alignments show an α_1_-NKA variant with residues like those present in CTS-susceptible α-NKA isoforms which this study refers to as α_1_A-NKA. A second variant shows substitutions previously related to CTS resistance and is referred herein as α_1_B-NKA. α_1_A-NKA and α_1_B-NKA variants presented in *A. minutus*, *P. aurotaenia*, and *E. boulengeri* have a percentage of identity of 98–99%. Five substitutions are shared by the three species, and most of these changes are concentrated between the TM1-2 helices (Table 4).

**Table 4:** Substitutions presented in the extracellular loops of α_1_ isoform variants identified in three species of dendrobatids collected in the Chocó Biogeographical Region, Valle del Cauca, Colombia

Species	Mismatches	Position	α_1_A	α_1_B
*P. aurotaenia*	7	103 (L1/2)	V	L
		106 (L1/2)	L	M
		111 (L1/2)	Q	R
		112 (L1/2)	A	S
		113 (L1/2)	A	V
		116 (L1/2)	E	D
		122 (L1/2)	N	D
*E. boulengeri*	5	106 (L1/2)	L	M
		111 (L1/2)	Q	R
		112 (L1/2)	A	S
		116 (L1/2)	E	D
		122 (L1/2)	N	D
*A. minutus*	7	102 (L1/2)	I	L
		106 (L1/2)	L	M
		111 (L1/2)	Q	R
		112 (L1/2)	A	S
		116 (L1/2)	E	D
		122 (L1/2)	N	D
		181 (L2/3)	G	A

### Amino Acids Substitutions in α_1_ Isoforms Identified in Skeletal Muscle Transcriptomes of Dendrobatid Species

**Fig. 2:**
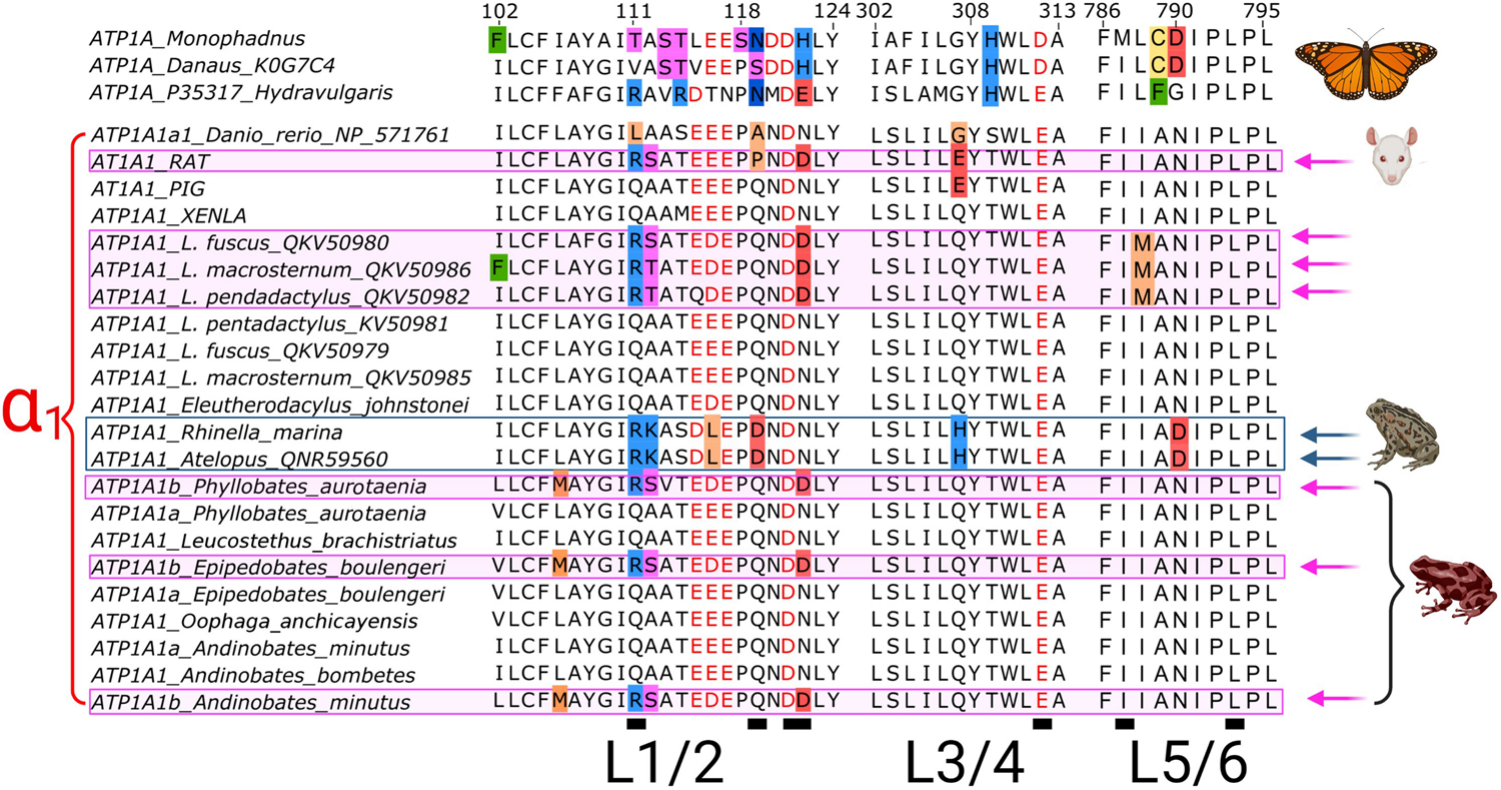
Alignments of the first three extracellular loops of the α_1_ isoform identified in the transcriptomes of six species of dendrobatids: *P. aurotaenia*, *O. anchicayensis*, *E. boulengeri*, *A. bombetes*, *A. minutus,* and *L. brachistriatus.* Isoforms with resistance substitutions are indicated in boxes. The fuchsia boxes show those isoforms characterized by substitutions L106M, Q111R, A112S and N122D. The blue box highlights bufonid species with substitutions Q111R, A112K, D/E116L, Q119D. Negatively charged amino acids are highlighted with red font color. The substitutions with respect to the susceptible phenotype (*S. scrofa*) found in each isoform are highlighted with small boxes filled with colors according to the properties of its side group: Aliphatic-nonpolar (orange), aromatic-nonpolar (green), negatively charged acid (red), positively charged basic (light blue), polar neutral-hydroxyl (pink), polar neutral-sulfur (yellow), polar neutral-amide (dark blue). Animal cartoons were taken from BioRender.com

α_1_A-NKA presents residues like those reported in CTS susceptible isoforms in species like *S. scrofa*, *E. johnstonei*, and *X. laevis*. In contrast, α_1_B-NKA identified in *P. aurotaenia*, *E. boulengeri*, and *A. minutus* transcriptomes, presented residues in three positions (Q111R, A112S, N122D) similar to those found in CTS resistant isoforms reported for leptodactylid frogs and in rodents (*Rattus norvegicus* and *Mus musculus*) (Price and Lingrel 1988; Mohammadi et al. 2021). A replacement of a leucine by a methionine (L106M) was also characteristic and unique of the α_1_B variant (Fig. 2).

### Amino Acid Substitutions in α_2_ Isoforms Identified in Skeletal Muscle Transcriptomes of Dendrobatid Species

**Fig. 3:**
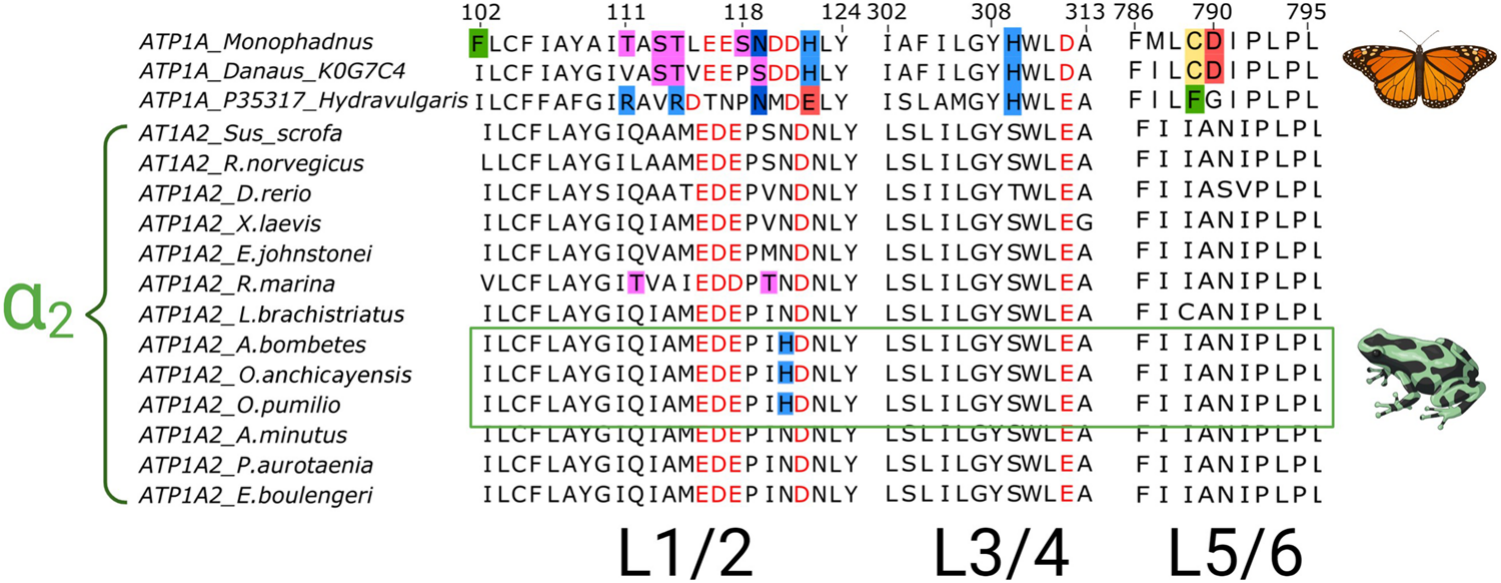
Alignments of the first three extracellular loops of α_2_ isoform identified in the transcriptomes of six dendrobatid species: *P. aurotaenia*, *O. anchicayensis*, *E. boulengeri*, *A. bombetes*, *A. minutus*, and *L. brachistriatus*. Isoforms with resistance substitutions are indicated in boxes**.** The green box highlights the α_2_ isoform with the N120H substitution. Negatively charged amino acids are highlighted with red font color. The dissimilarities with respect to the susceptible phenotype *(S. scrofa, X. laevis, E. johnstonei)* found in each isoform are highlighted with small boxes colored according to the properties of its side chain: Aliphatic-nonpolar (orange), aromatic-nonpolar (green), negatively charged-acid (red), positively charged basic (light blue), polar neutral-hydroxyl (pink), polar neutral-sulfur (yellow), polar neutral-amide (dark blue). Animal cartoons were taken from BioRender.com

The NKA isoform identified as α_2_ in *P. aurotaenia*, *E. boulengeri*, and *A. minutus* does not contain substitutions associated to CTS-resistance. In contrast, *A. bombetes* and *O. anchicayensis* presented an α_2-_NKA with one substitution at the position 120, where an asparagine is replaced by a histidine (N120H) (Fig. 3). This substitution was not only observed in these species, but also for one of the α isoforms (identified as α_2_ by Blast analysis) found in assembled transcriptomes for *Oophaga pumilio* published in the TSA/NCBI database. These transcriptomes were obtained from individuals collected in the Bocas del Toro archipelago in Panama (Access Code: GIKS01400520; BioProject: PRJNA610154). This isoform shared 100% identity at the protein level with respect to the sequence found in *O. anchicayensis.*

**Fig. 4:**
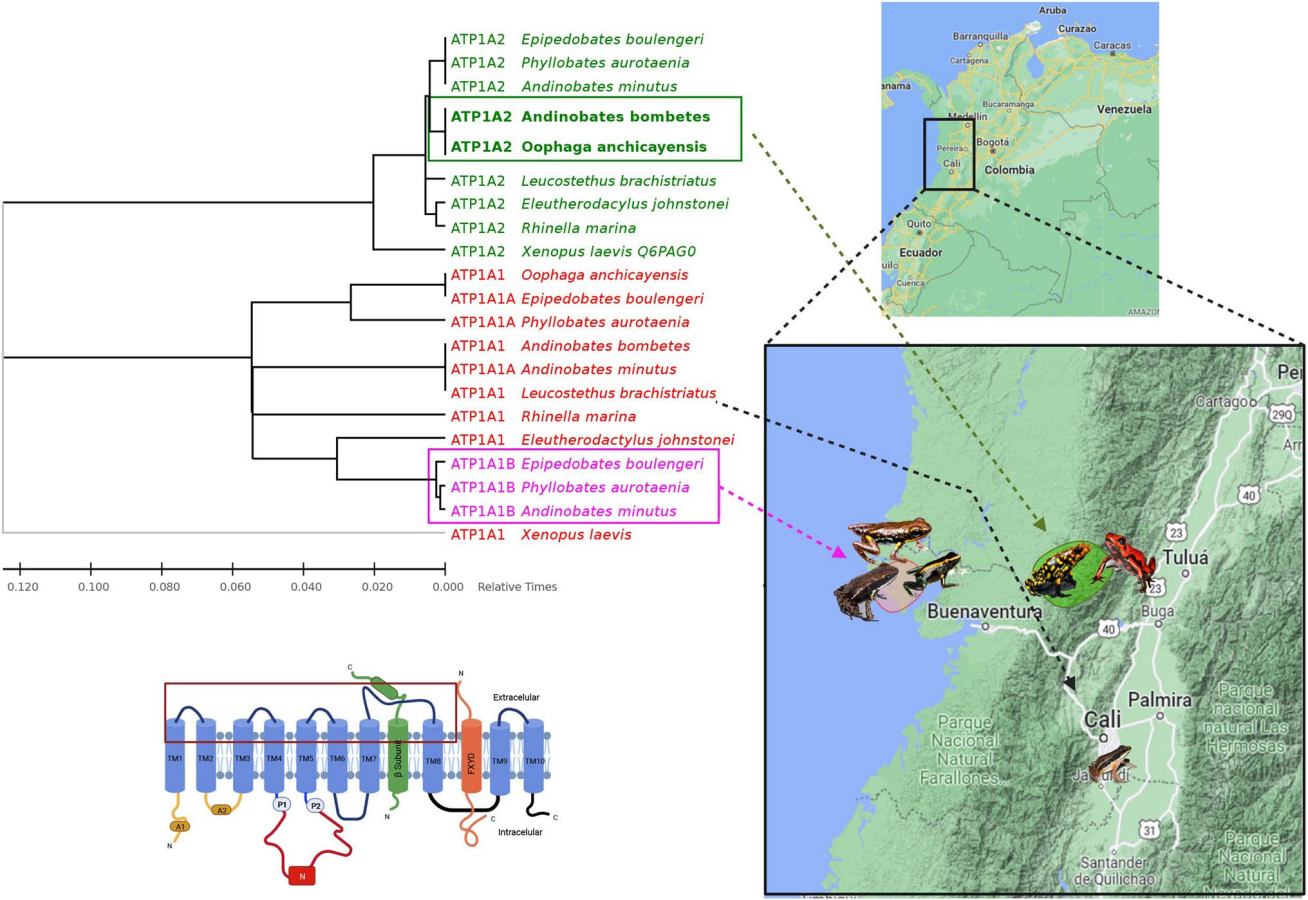
Phylogenetic tree created with the extracellular loops involved in CTS resistance phenotypes. The boxes show the isoforms with CTS resistance substitutions and the geographic distribution of the species that contain them. The time tree shown was generated using the RelTime method (Tamura et al. 2012). Divergence times for all branch points in the topology were calculated using the Maximum Likelihood method and the Le_Gascuel_2008 model (Le and Gascuel 2008). The estimated log-likelihood value of the topology shown is -591.61. A discrete Gamma distribution was used to model the differences in evolutionary rates between sites (5 categories (+ G, parameter = 0.2666)). The tree is drawn to scale, with branch lengths measured in the relative number of substitutions per site. This analysis involved 22 amino acid sequences. There was a total of 96 positions in the final data set. Evolutionary analyses were performed on MEGA11 (Tamura et al. 2021)

The phylogenetic tree shown in Fig. 4 clusters the amino acid sequences encoding the first four extracellular loops in each paralog. This clustering shows the shared substitutions implicated in low CTS affinity among species from different genera seems to have an influence from their geographical distribution.

### Molecular Docking and BE Trends

**Fig. 5:**
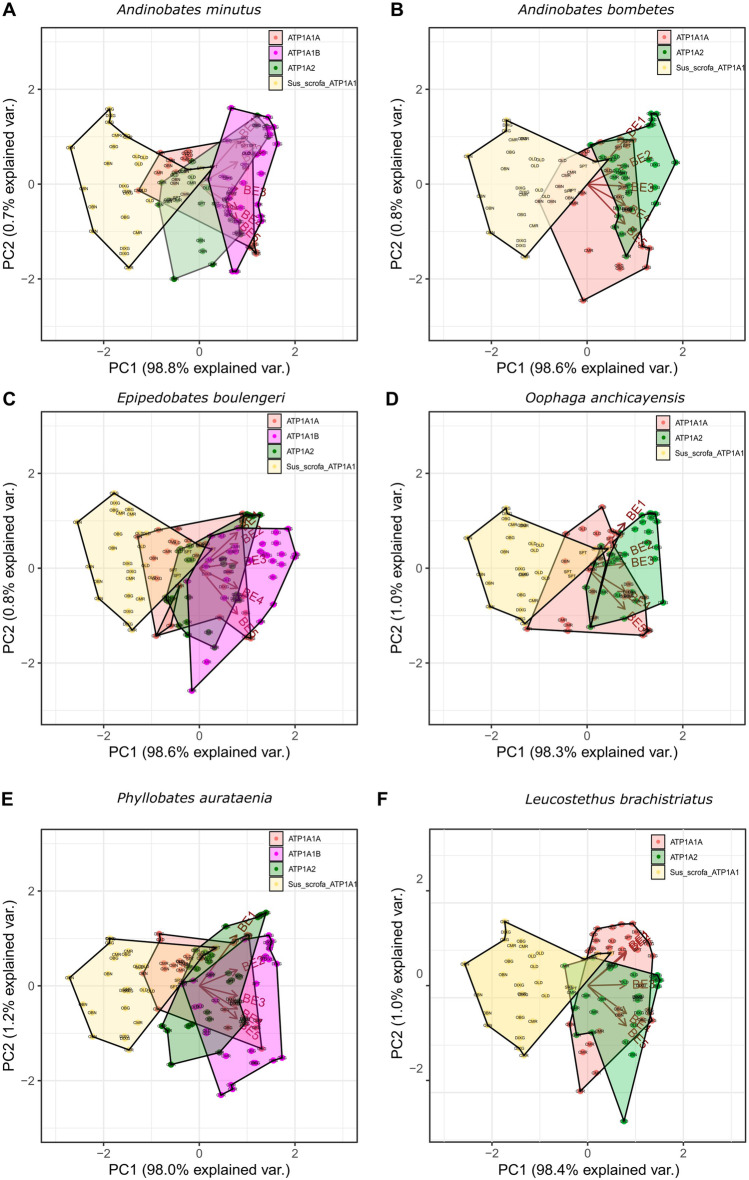
Principal component analysis (PCA) for binding energy (BE) estimated for different CTS-NKA α complexes for Dendrobatid species. Each receptor α is represented as a convex hull, which are areas circumscribed by points; each dot represents the binding energy (BE) for a specific αNKA-CTS complex. Receptors for each species: ATP1A1A (α_1_A, isoform without resistance substitutions), ATP1A1B (α_1_B isoform with resistance substitutions), ATP1A2 (green α_2_ isoform) were plotted together with the α_1_ isoform of *S. scrofa* (in yellow). **A**, **C**, and **E** species collected in Bahía Málaga, Buenaventura, Chocó Biogeographical Region, Colombia. Species **B** and **D** collected in the municipalities of Yotoco and Dagua on the Western Cordillera. Species **F**, Collected in the Valle del Rio Cauca, in the municipality of Santiago de Cali. The clusters of αNKA-CTS complexes with the highest affinity are distributed towards the left of the plane, and the clusters of αNKA-CTS complexes with the lowest affinity are located towards the right

PCA highlights and clusters patterns of affinity based on the binding energies (BE) values ​​of each receptor with the selected CTS toxins. The five lowest BE values (measured in kcal/mol), BE1-BE5, obtained from the molecular docking of each α-NKA-CTS complex (receptor-toxin) were selected for this analysis (Suplemental Dataset 2). There is a greater dispersion of the data on the first principal component (PC1, horizontal axis; Fig. 5A-F). This component explains ~ 98.0–98.8% of the variance of the data, whereas the second principal component (PC2, vertical axis) accounts for only 0.7–1.2%. Therefore, variations in affinity are distributed along the horizontal axis. The NKA α isoforms from dendrobatid frogs with the susceptible phenotype are located between *S. scrofa* complexes and the isoform with the resistant phenotype, validating the predictions based on the amino acid sequences.

**Fig. 6:**
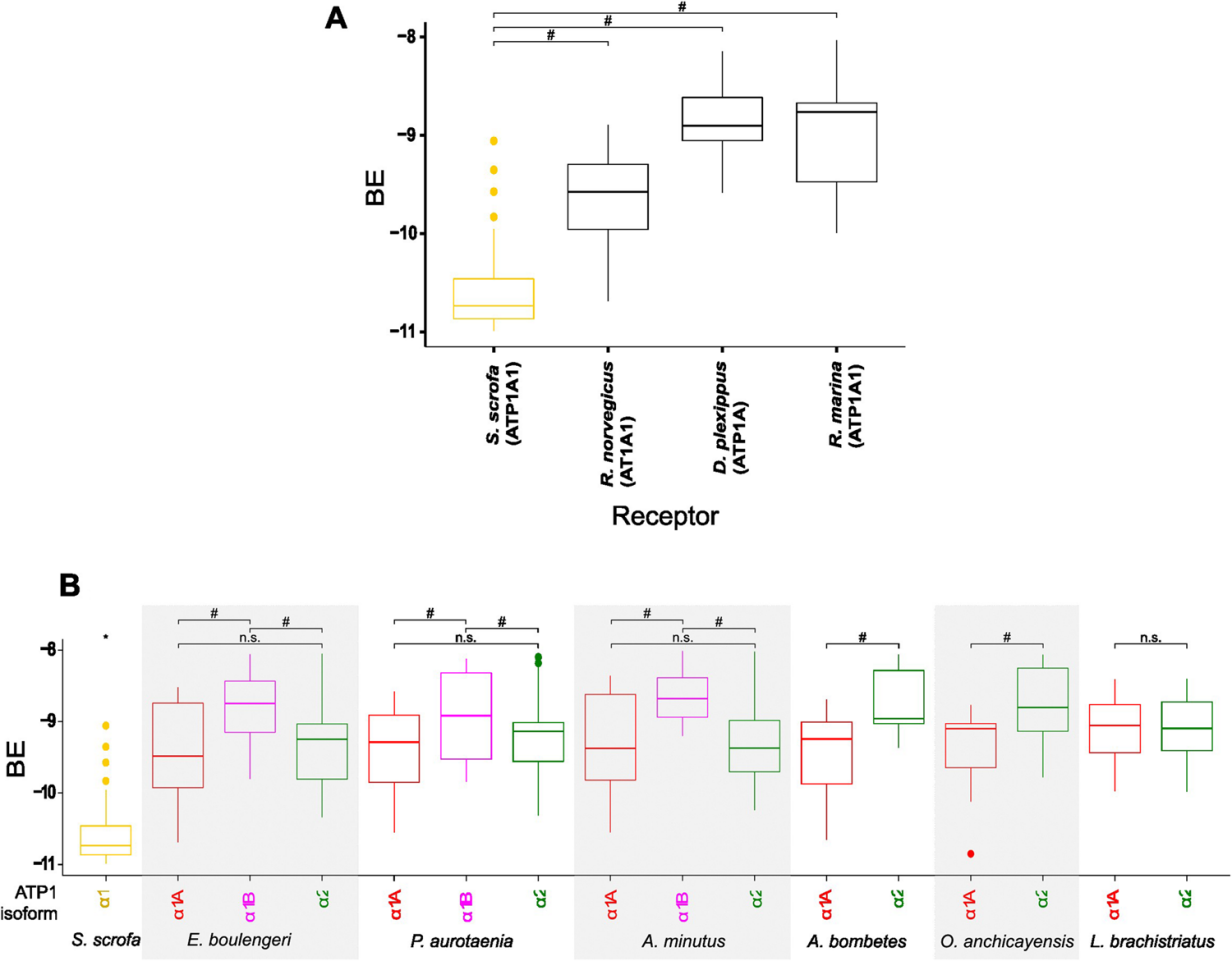
Box plot of the best docking scores obtained for each receptor from its interaction with six cardenolide-type cardiotonic steroids (CTS). **A** α-Isoforms whose affinity for CTS has been functionally demonstrated, ATP1A1 from *Sus scrofa*, ATP1A1 from *Rattus norvegicus*, ATP1A1 from *Danaus plexippus*, ATP1A1 from *Rhinella marina*. **B** Identified α- NKA isoforms for dendrobatid species modeled by homology: *E. boulengeri*, *P. aurotaenia*, *A. minutus*, *A. bombetes*, *O. anchicayensis*, *L. brachistriatus.* The pig α_1_ isoform (ATP1A1 from *S. scrofa*) is used as a control, considered to be the isoform with the highest susceptibility. The horizontal line in the middle of the boxes corresponds to the mean of the data (*n* = 5, molecular docking was run five times). #: *p* < 0.05 post-hoc Tukey mean comparison test between receptors in each species α_1_A vs. α_1_B, α_1_A vs. α_2_, and α_1_B vs. α_2_. *: *p* < 0.05 All α_1_ complexes are statistically different from *S. scrofa (*control)

The BE estimates for the best poses for six different CTSs on their α-NKA binding pocket in each isoform are shown in Fig. 6. For comparative purposes, the complexes created for the *S. scrofa* α_1_ receptor (PDB: 7EVX) characterized by having high affinity for CTS were included, as well complexes created by homology modelling using *Rhinella marina*, *Rattus norvegicus*, and *Danaus plexippus* α_1_ isoforms as receptors, whose low affinity for CTS has been functionally confirmed (Gable et al. 2017; Jaisser et al. 1992; Petschenka et al. 2013). These receptors used as references were docked to six types of CTS and showed a significant reduction (*P* < 0.05) in their affinity compared to the control of *S. scrofa* α_1_-NKA. This has been observed at the functional level, supporting the *in-silico* approach used in this work to evaluate resistance in new isoforms (Fig. 6A; Table S2-S4). Comparative analysis between isoforms with and without resistance substitutions within the same species showed significant differences for the BE (Fig. 6B). All receptors created from the amino acid sequence for α-NKA isoforms of dendrobatid frogs (including those that retain the susceptible state) showed a significant reduction in affinity for CTS compared to the mammal control (Table S5).

## Discussion

The bioaccumulation of alkaloid-type toxins in poison dart frogs of the Dendrobatidae family is well documented; however, information regarding possible exposure to CTS is practically non-existent. Analysis of skeletal muscle transcriptomes from six species representing five genera of the Dendrobatidae family allow us to identify two different α-NKA isoforms: α_1_ and α_2_-NKA. All the isoforms identified showed similar length to their homologous counterparts in other anurans. The species *L. brachistriatus*, *O.anchicayensis*, and *A. bombetes* were characterized as presenting a single variant for each of the two isoforms. In contrast, two potentially coding variants for the α_1_-NKA isoform and one single variant for α_2_-NKA isoform were found in skeletal muscle transcriptomes from *P. aurotaenia*, *E. boulengeri*, and *A. minutus*. Indeed, the results show that the transmembrane segments and the loops L3/4 and L5/6 are highly conserved in both α_1_-NKA and α_2_-NKA. On the other hand, the main differences identified between the two α_1_-NKA variants occurred at the loop L1/2*.* The identification of these sequences provided important insights about the evolutionary relationships and origin of these αNKA with respect to their homologous counterparts. In addition, protein homology alignments predicted, based on previous research and the biochemical properties of the residues, how these substitutions might affect the affinity of these new α-NKA sequences for CTS. In silico analyses supported these predictions.

### α_1_-NKA Isoforms in Dendrobatids Frogs and their Phylogenetic Relationships

As revealed by the results, *P. aurotaenia, E. boulengeri*, and *A. minutus* presented two different variants for the α_1_-NKA isoform, one α_1_B with Q111R/N122D (resistant phenotype), and a α_1_A-NKA and α_2_-NKA isoform that conserves the susceptible state. Independent events of gene duplications encoding CTS-susceptible and CTS-resistant phenotypes have been published for multiple species within Insecta, including the families Coleoptera, Hemiptera, and Diptera (Lohr et al. 2017; Petschenka et al. 2017; Yang et al. 2019; Zhen et al. 2012). In vertebrates, there are reports of these events only for anurans of the genus *Leptodactylus* and two species of the dendrobatids, *Oophaga pumilio* and *Ranitomeya imitator* (Hernández Poveda 2022; Mohammadi et al. 2022a; Mohammadi et al. 2021; Moore et al. 2009).

Phylogenetic studies for four of the major species groups within *Leptodactylus* by Mohammadi et al. (Mohammadi et al. 2021) showed that the tree topology estimated from full gene sequences suggest an independent duplication followed by parallel substitutions; In contrast, the tree topology obtained from amino acid sequences supports that the duplicate variants found in *Leptodactylus* species occurred in the most recent common ancestor of the genus. These authors proposed that the phylogeny that shows a common origin is more parsimonious, and the differences observed in the topologies may be due to non-allelic gene conversion between duplicated genes that causes a homogenizing effect in both loci, and cluster in the phylogenetic analysis both paralogs together (Mohammadi et al. 2021).

The protein tree shown in Fig. 1 and the one inferred from nucleotides (Fig. S1) for dendrobatid sequences had a similar topology to the nucleotide phylogeny presented by Mohammadi et al. (2021). This protein phylogenetic tree is also largely consistent with those obtained in phylogenetic analyses of nuclear and mitochondrial genomic markers (Guillory et al. 2019) and both nuclear and mitochondrial genomic markers and morphological data (Grant et al. 2017). In the α_1_-NKA isoform clade, *Phyllobates* is sister to the other genera that form the subfamily Dendrobatidae, which includes *Oophaga* and *Andinobates*, and *Leucostethus* is grouped with *Epipedobates* in Colostethinae. The presence of species with one and two α_1_-NKA variants coding for susceptible and resistant phenotypes in both subfamilies supports the hypothesis that this event could have preceded the diversification of the dendrobatid clade into separate genera and species. In this case, this feature might be preserved by positive selection in some species and in others has been lost by a decrease in selective pressure. Hernandez-Poveda et al. (2022) proposed that the similarity presented between resistant and susceptible α_1_-NKA sequences within dendrobatid species, in comparison with their homologous in other species, suggests similar pattern of selection between duplicated genes to the one presented in *Leptodacylus* (which follows a model of concerted evolution between duplicate genes and purifying selection acting on the CTS resistance sites). To evaluate this hypothesis, however, robust phylogenetic analysis at the genomic level is needed.

### Amino Acid Substitutions at Sites Implicated in CTS Resistance and CTS Binding in α-NKA Isoforms

The CTS binding pocket has been well characterized both structurally, by crystallography, and functionally, by combining site-directed mutagenesis, biochemical, electrophysiological techniques (Croyle et al. 1997; Kanai et al. 2021; Kanai et al. 2013; Lingrel et al. 1991; Mohammadi et al. 2022a; Ogawa et al. 2009; Price and Lingrel 1988). Along with functional experiments, protein homology comparisons and phylogenetic studies have shown that substitutions found in the L1/2 extracellular loop are responsible for the differential CTS affinity among paralogous and orthologous isoforms within and between species (Mohammadi et al. 2022a). Consistent with these studies, the extracellular L1/2 loop has most of the variability among poison dart frog α-NKA isoforms (Fig. 2).

According to the positions and residues related to the binding of CTS on the basis of the latest NKA crystals (Kanai et al. 2021), and the substitutions previously implicated in CTS susceptibility through experimentation *in vitro* (residues summarized by Yang et al. (2019)), this study identified structural changes in the amino acids sequence of α-NKA isoforms in skeletal muscle of six species of dendrobatids. Only the αB-NKA variant found in *P. aurotaenia*, *E. boulengeri*, and *A. minutus,* seems to have substitutions involved in resistance in the CTS binding pocket. The structure and nature of four of these substitutions, Q111R, A112S, N122D, and L106M (Fig. 2), could explain the low affinity obtained in the molecular docking results (Fig. 6). As mentioned in the results, Q111R, A112S and N122D are also present in the CTS-α_1_NKA resistant and functionally characterized isoform from *Rattus norvegicus*, based on this, previous structural studies have predicted the possible formation of a salt bridge between charged residues (i.e., between Q111R and N122D) at both ends of this extracellular loop as the main responsible of a reduced affinity (Kanai et al. 2021). In the case of L106M, although methionine is an aliphatic residue and is not often considered to form hydrogen bonds, there are examples where methionine serves as an H-bond acceptor with backbone amides (Biswal and Wategaonkar 2009). It has also been reported methionine residues can interact with aromatic amino acids through non-covalent bonds causing new molecular interactions to nearby amino acid residues (Weber and Warren 2019). These two types of intermolecular forces in the NKA binding cavity could constrain the CTS bond.

**Fig. 7:**
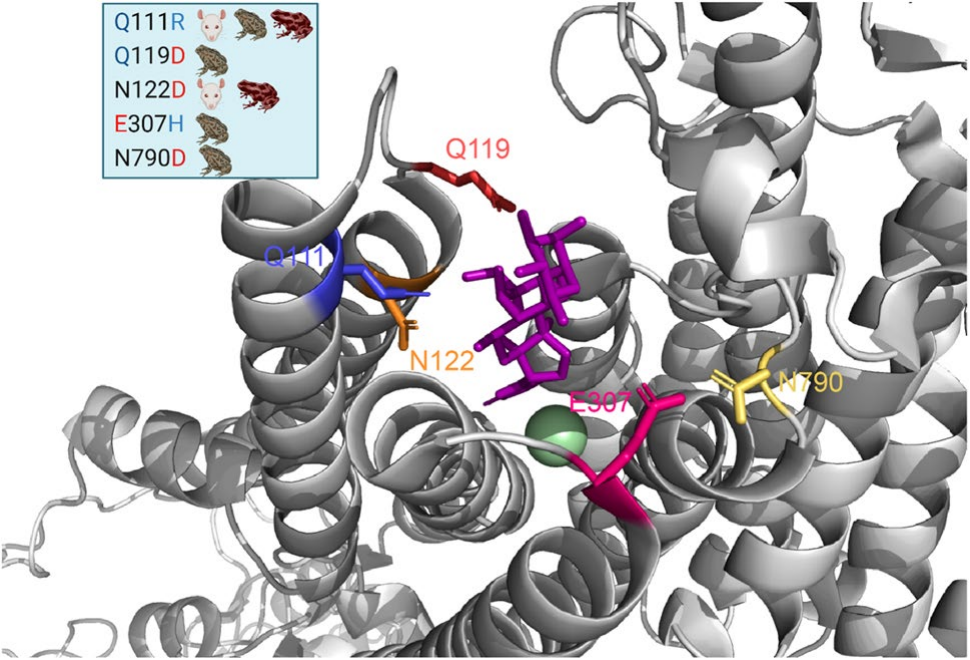
Positions of residues responsible for resistance to CTS. The figure shows the CTS Binding cavity in the NKA of the *S. scrofa* crystal, the residues in this CTS phenotype are highlighted with different colors. The blue box on the top left lists some of the substitutions found in these positions in different species. Animal cartoons were taken from BioRender.com

In bufonid anurans only a single variant for each paralog has been reported (Medina-Ortiz et al. 2021; Mohammadi et al. 2022a; Moore et al. 2009). The bufonid α_1_-NKA isoform presents four substitutions in the L1/2 loop, Q111R, A112K, E116L, Q119D (Fig. 2). Bufonids also feature substitutions at E/Q307H and N790D in the loops L3/4 and L5/6 respectively, which are not present in dendrobatid α_1_-NKA isoforms. These two unique mutations are in the vicinity of E312 and R880 (L7/8), two residues involved in OBN binding, and could cause the formation of salt bridges with either of these charged residues, constraining the binding of CTS even more (Fig. 7).

Recent evolutionary studies of NKA show that the α_1_ paralog exhibits the greatest interspecific variation involved in CTS resistance, presenting most of the substitutions at the positions 111 and 122, whereas resistance substitutions in α_2_ and α_3_ have been reported mainly in reptiles and birds, with changes predominantly at positions 111 and 120 (Mohammadi et al. 2022a). Our study presents the first report of a N120H substitution for the α_2_ isoform in anurans. A substitution of an asparagine for a histidine at the C-terminal of this extracellular loop, at the position 122, has convergently evolved in at least five orders of insects that consume and/or sequester CTS (Dobler et al. 2015; Petschenka et al. 2017). Mutations by positively charged residues at position 120 in tetrapod vertebrates (e.g., N/G120R in reptiles and amphibians) for α_2_ and α_3_ have been previously reported (Mohammadi et al. 2016; Mohammadi et al. 2022a; Ujvari et al. 2015). A reduction of affinity to CTS by histidine and arginine substitution at the C-terminal of L1/2 has been confirmed experimentally in both invertebrates and vertebrates’ isoforms (Dalla and Dobler 2016; Dalla et al. 2013; Dobler et al. 2012; Mohammadi et al. 2022a; Ujvari et al. 2013). Although a reduction in affinity to CTS for N120H containing α_2-_NKA isoforms has not been confirmed by *in vitro* experimentation, this study suggests that the presence of this residue could have a similar effect to H122 reported for insects and vertebrates. The introduction of basic residues in a cavity characterized by being negatively charged due to the presence of acid residues D/E (6 residues of this type in *S. scrofa*) would promote ionic or polar interactions, which could constrain CTS binding. In addition, the bulky side chain of histidine could be a steric obstacle for the CTS bond.

The recurrence of certain patterns of substitutions flanking the L1/2 loop in some lineages and paralogs could reflect bias linked to mutational and physicochemical constraints. Molecular evolution studies have shown that proteins evolve under many biophysical selection pressures that collectively determine the patterns of amino acid substitutions (Norn et al. 2021). Modifications at the structural level constitute a loss of protein stability and a large energy cost, whereby most proteins can tolerate only small changes (Sikosek and Chan 2014). Therefore, L1/2 loop constitutes the most thermodynamically favorable site for the accumulation of mutations associated with resistance to CTS, since other sites outside this loop could lead to impairment of the functional activity of the NKA (Dalla and Dobler 2016; Petschenka et al. 2017). This would explain the evolutionary convergence and parallelism observed in different lineages under the same selective pressure.

### Reduced Number of Substitutions between α_1_-NKA Duplicates Could Indicate Different Compensatory Mechanism for Pleiotropic Effects

The presence of resistance conferring substitutions at the first extracellular loop could result in deleterious pleiotropic effects such as reduced NKA activity or altered cation affinity (Dalla et al. 2017; Dobler et al. 2012). Mohammadi et al. (2021) observed that some wildtype resistant NKAs possess substitutions distributed along their coding sequence that restore the loss of function caused by resistant mutations; however, this compensatory mechanism is not pervasive across species (Dalla and Dobler 2016; Mohammadi et al. 2022a; Petschenka et al. 2017). Most of the differences (5–7 substitutions) between the susceptible α_1_A and the resistant α_1_B variant (within the same species) identified in A. *minutus, P. aurotaenia*, and *E. boulengeri* transcriptomes are located between the first two transmembrane segments, the rest of the amino acid sequence is identical between both variants. This could suggest that fewer substitutions in these isoforms could be playing a similar compensatory role in dendrobatids as the one described by Mohammadi et al. (2021) for Leptodactylus, or maybe there is another compensatory mechanism that do not depend on the sequence itself that could mitigate the pleiotropic effects associated for containing isoforms with resistant substitutions. An alternative hypothesis could be the presence of a regulatory mechanism linked to the level of expressions of both isoforms, which could explain why some anuran species still conserved the susceptible form. It is also possible that multiple mechanisms may work together to counteract any negative pleiotropic effects.

Previous studies have observed that resistant substitutions together with the levels of expression of different paralogs play an important role in the overall resistance to CTS and physiological role of each tissue (Mohammadi et al. 2018; Yang et al. 2019). In addition, studies in human α-NKA isoforms have shown that a reduction of the level of expression of some isoforms might be involved in an increase in susceptibility to CTS (Wang et al. 2001). Based on these, in addition to the expression of resistant isoform as a mechanism to cope with CTS exposure, high expression of a susceptible isoforms would enable maintenance of physiological homeostasis in each tissue. Although the estimation of relative abundance in each tissue provides insights about the level of expression of each isoform (Fig. S2) in these tissues, this type of analyses is exploratory and more robust studies of gene expression together functional assays are needed to proof the hypothesis proposed.

### Diversity of α_1_-NKA and α_2_-NKA Isoforms with Different CTS Resistant Substitutions in Skeletal Muscle of Dendrobatids from Different Localities

Intriguingly, this study observed that species from different localities contain CTS resistant substitutions in different α-NKA paralogs. *P. aurotaenia*, *E. boulengeri*, and *A. minutus*, species collected in the Chocó Biogeographical Region, despite being of different genera, showed the same transcriptional diversity for αNKA isoforms: two different variants for the α_1_-NKA isoform, one α_1_B with Q111R/N122D (resistant phenotype), and a α_1_A-NKA and α_2_-NKA isoform that conserves the susceptible state. In contrast, *O. anchicayensis* and *A. bombetes* collected on the Western Cordillera presented only one single α_1_ without resistance substitutions, and one α_2_ with a N120H substitution that could potentially translate for a resistant phenotype*. L. brachistriatus,* collected to the south of the city of Cali was characterized as presenting the susceptible phenotype in both identified isoforms, α_1_ (111Q/122N) and α_2_ (120N). It is worth noting that although the observed differences in the distribution of these isoforms among species could indicate the influence of geographical factors, it is possible that the expression of isoforms with substitutions different from those observed may also occur. Population-level genomic and transcriptomic analyses of conspecific species from different localities or closely related species from the same location can provide insights into the evolutionary forces influencing the distribution of these substitutions.

A well-known example of geographical patterns of resistance have been observed in populations of the snake *Thamnophis sirtalis* and its toxic prey, the newt *Taricha granulosa*, whose co-evolution has resulted in a variety of phenotypes resistant to tetradotoxin (TTX) resulting from sympatric exposure. The toxicity in newts and resistant of snakes exhbitis a geographic mosaic of levels of TTX and snake resistant, in which some regions feature highly toxic newts and similarly resistant snakes, while others feature less toxic newts and less resistant snakes (Brodie and Brodie 1991; Brodie et al. 2002). The variations in resistance in these populations are related both to the type of substitutions and to the expression levels of the different paralogs in the Na_V_1 sodium channel family, which are targets of these toxins (Brodie and Brodie 2015; Brunet et al. 2005; Feldman et al. 2016; Hague et al. 2017; McGlothlin et al. 2014). These studies have provided important insights about the resistance patterns of amino acid substitutions and their correlation with geographic distribution. Similar approaches could be applied to the study of CTS-resistant susbstitutions in dendrobatid populations.

### Possible Functional Consequences of CTS -Resistant α-NKA Isoforms in Skeletal Muscle

In mammalian skeletal muscle cells, the excitability is dependent on the functional activity and expression levels of the α_1_ and α_2_ isoforms (Clausen 2013; Pirkmajer and Chibalin 2016). The absence of the α_2_ isoform in skeletal muscle rapidly produces fatigue, and the overexpression of α_1_ does not compensate for the loss of function of α_2,_ which shows the importance of both isoforms in the overall function of skeletal muscle. α_2_ is capable of dynamically adapting to fluctuating Na^+^ and K^+^ transport demands in periods of excitable activity (Pirkmajer and Chibalin 2016).

The functional role of the NKA in skeletal muscle cells is also regulated by ECTS. It has been demonstrated that the α_2_ isoform in mouse skeletal muscle is regulated dynamically by an endogenous ligand acting on the cardiac glycoside-binding site, and it was proposed that this mechanism can play a physiological role in dynamic adaptations to exercise (Radzyukevich et al. 2009). More recently it has been shown that circulating OBN regulates rat skeletal muscle electrogenesis by increasing the α_2_ isoform transport activity (Kravtsova et al. 2020). On the other hand, other cellular functions could be mediated by the skeletal muscle NKA. For example, it has been demonstrated in human skeletal muscle that the CTSs OBN and marinobufagenin increase glycogen synthesis (Kotova et al. 2006). Additionally, it has been shown that OBN suppresses the IL-6/STAT3 signaling but promotes secretion of IL-6 and other cytokines in cultured human skeletal muscle cells (Pirkmajer et al. 2020). Although the receptor was not identified, in both cases it was proposed to be the NKA. In agreement with this evidence and considering that the levels of ECTS are not above 1 nM, Orlov et al. (2021) proposed that low doses (below 1 nM) of ECTS activate NKA, promoting cell proliferation. They also proposed that these low doses of ECTS stimulate NKA conformational changes that activate kinases and Ca^2+^ oscillations. In contrast, doses above 1 nM will inhibit NKA, promoting gene expression, protein synthesis, and cell adhesion. Although the presence of ECTS has not been reported in dendrobatids, we cannot rule out their presence or the possibility that they modulate the activity of the NKA isoforms that we identified. This summarize all potential functions (assuming homologous NKA regulatory mechanisms in amphibians) that can be affected either for the inhibition of susceptible paralogs for the NKA expressed in skeletal muscle or for the presence of CTS resistant forms in these tissues (which would inhibit the role of the NKA as a signal receptor).

As it was mentioned above, the introduction of residues that confer resistance to CTS in the first extracellular loop causes a reduction not only in the affinity for OBN, but also in the cationic affinity and ATPase function, in addition to the possible negative effect on the role of NKA as a signal receptor (Lingrel 2010). It has been hypothesized that preservation of the susceptible phenotype, resistance substitutions linked to specific paralogs, and compensatory substitutions in other regions of the primary NKA structure could buffer the negative physiological consequences and energy costs of having resistant isoforms. Except for *L. brachistriatus*, all the other species of Dendrobatidae presented at least one transcript encoding one α isoform with substitutions characteristic of a CTS resistant phenotype. Molecular docking analysis supports resistance prediction related to CTS sustitutions by showing BEs with less negative values for these isoforms with resistant substitutions compared to their susceptible counterparts. Future research at different biological levels could evaluate the physiological outcomes of having resistant and susceptible isoforms (and different variation of their level of expression) by implementing a combination of molecular biology (i.e., mutagenesis, chimeras, knock-down, knock-out), biochemical (ATPase assays), and electrophysiological (voltage clamp) approaches.

The evolution of the isoforms and the CTS resistant substitutions found in α-NKA isoforms from dendrobatids could have been influenced by biophysical, physiological, and geographical constraints. However, to validate this hypothesis more research on this topic is needed. The results in this study suggest potential avenues for further research on dendrobatids in a variety of fields, including evolution, biophysics, chemical ecology, molecular ecology, physiology, and pharmacology.

## Supplementary Information

Below is the link to the electronic supplementary material.

Supplementary file1 (JPG 100 KB)

Supplementary file2 (JPG 218 KB)

Supplementary file3 (DOCX 184 KB)

Supplementary file4 (XLSX 172 KB)

Supplementary file5 (XLSX 26098 KB)

## Data Availability

**Table:** 

*NKA isoform*	*Species*	*Accession number* *GenBank*
*α*_*2*_	*Phyllobates aurotaenia*	OP006565
*α*_*1*_ *B*	*Phyllobates aurotaenia*	OP006566
*α*_*1*_ *A*	*Phyllobates aurotaenia*	OP006567
*α*_*2*_	*Oophaga anchicayensis*	OP006568
*α*_*1*_	*Oophaga anchicayensis*	OP006569
*α*_*1*_ *A*	*Andinobates minutus*	OP006570
*α*_*1*_ *B*	*Andinobates minutus*	OP006571
*α*_*2*_	*Andinobates minutus*	OP006572
*α*_*1*_	*Andinobates bombetes*	OP006573
*α*_*2*_	*Andinobates bombetes*	OP006574
*α*_*1*_	*Epipedobates boulengeri*	OP006575
*α*_*1*_ *B*	*Epipedobates boulengeri*	OP006576
*α*_*2*_	*Epipedobates boulengeri*	OP006577
*α*_*2*_	*Leucostethus brachistriatus*	OP006578
*α*_*1*_	*Leucostethus brachistriatus*	OP006579
