# Supplementary figures and images for: Identification of the NA+/K+-ATPase α-Isoforms in Six Species of Poison Dart Frogs and their Sensitivity to Cardiotonic Steroids
Source: J Chem Ecol. 2023 Mar 6;49(3-4):116–32. doi: 10.1007/s10886-023-01404-7 (PMC10102066; doi:10.1007/s10886-023-01404-7)

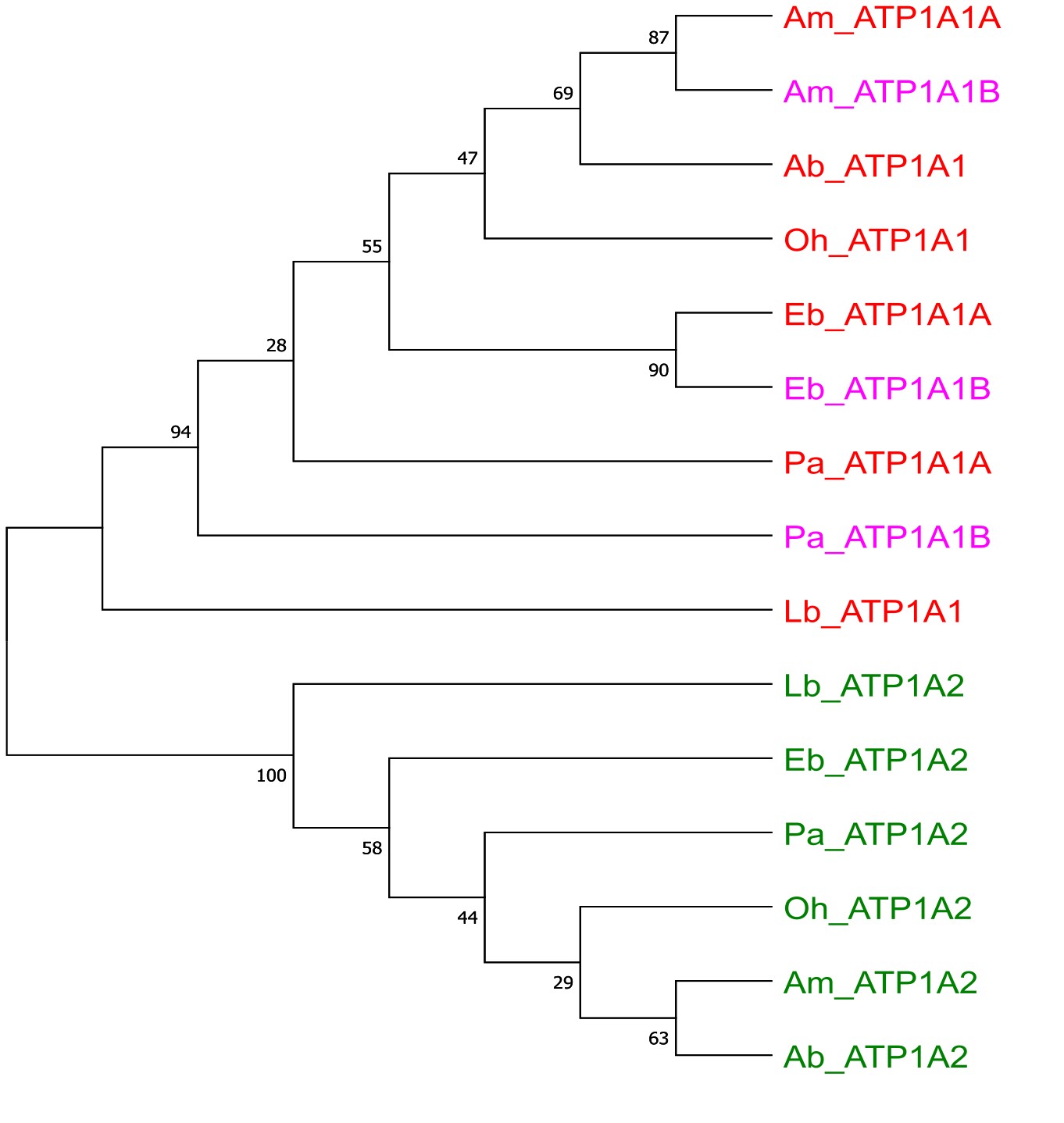

Supplement: Supplementary file 1 — Supplementary file1 (JPG 100 KB) [file 10886_2023_1404_MOESM1_ESM.jpg]

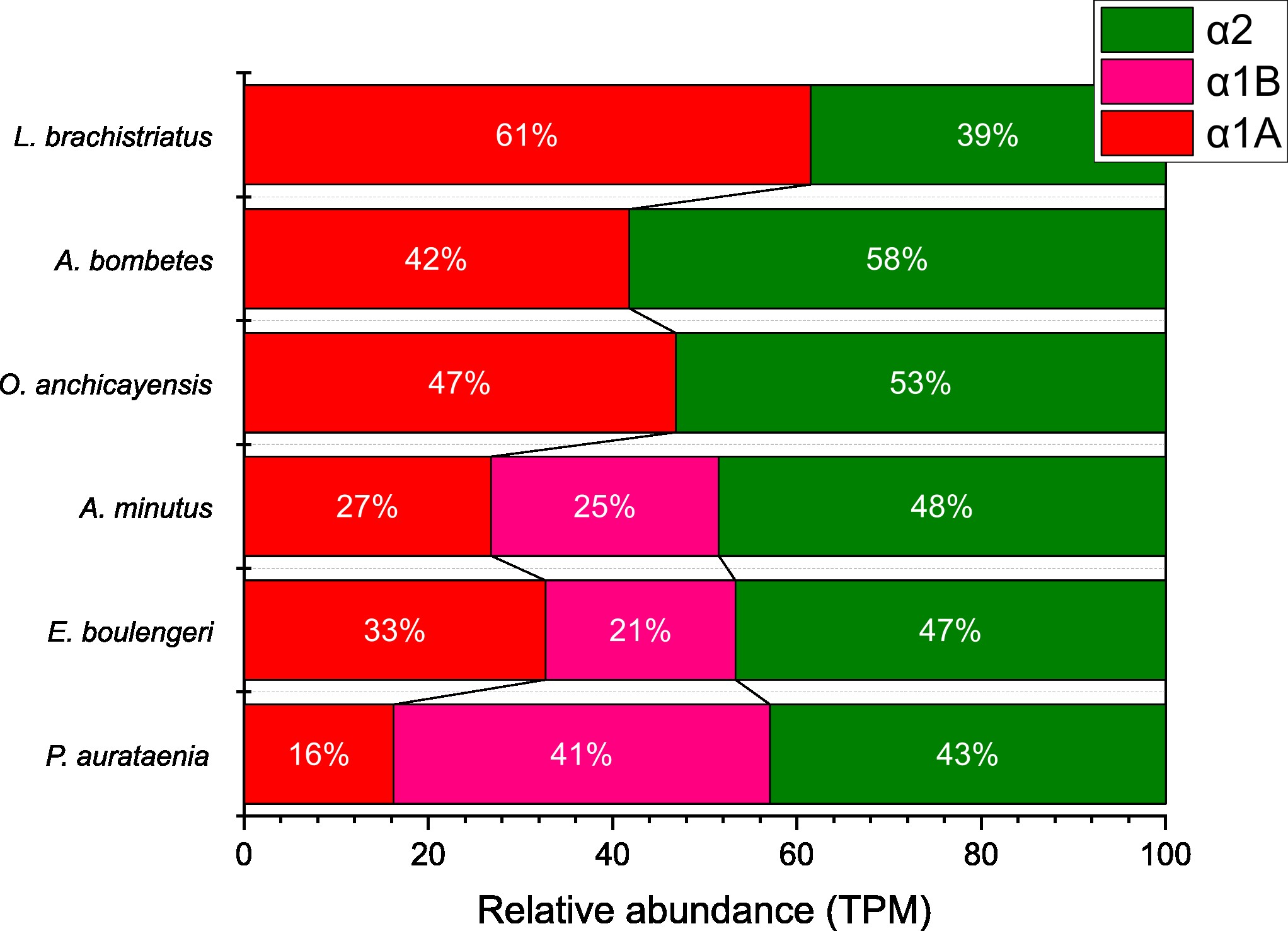

Supplement: Supplementary file 2 — Supplementary file2 (JPG 218 KB) [file 10886_2023_1404_MOESM2_ESM.jpg]
