# Supplementary material for: Identification of the NA+/K+-ATPase α-Isoforms in Six Species of Poison Dart Frogs and their Sensitivity to Cardiotonic Steroids
Source: J Chem Ecol. 2023 Mar 6;49(3-4):116–32. doi: 10.1007/s10886-023-01404-7 (PMC10102066; doi:10.1007/s10886-023-01404-7)
Supplement: Supplementary file 3 — Supplementary file3 (DOCX 184 KB) [file 10886_2023_1404_MOESM3_ESM.docx]

**Supplementary data**


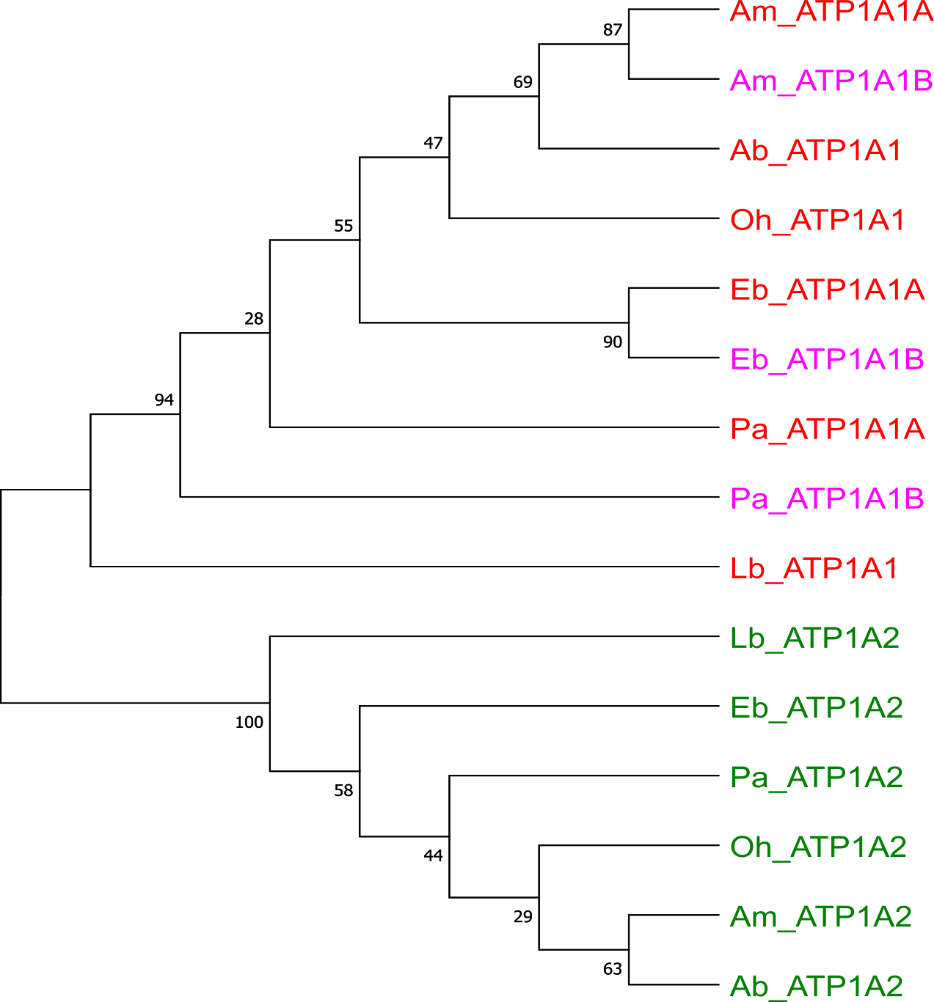


**Figure S1.** Phylogenetic tree constructed with cDNA sequences encoding α-NKA isoforms (CDS regions) from dendrobatid species. The tree was inferred by using the Maximum Likelihood method and Tamura-Nei model (TN93+G+I; 15 nucleotide sequences and 3075 positions). The tree with the highest log likelihood (-17783.57) is shown. The percentage of trees in which the associated taxa clustered together is shown next to the branches. Text colors: α_1_-NKA and α_1_A-NKA (ATP1A1) in red, α_1_B-NKA (ATP1A1B) in pink, α_2_-NKA (ATP1A2) in green. Am: *Andinobates minutus*; Pa: *P. aurotaenia*; Ab: *A. bombetes*; *E. boulengeri*; Am: *A. minutus;* Oh: *O. anchicayensis;* Lb: *L. brachistriatus*


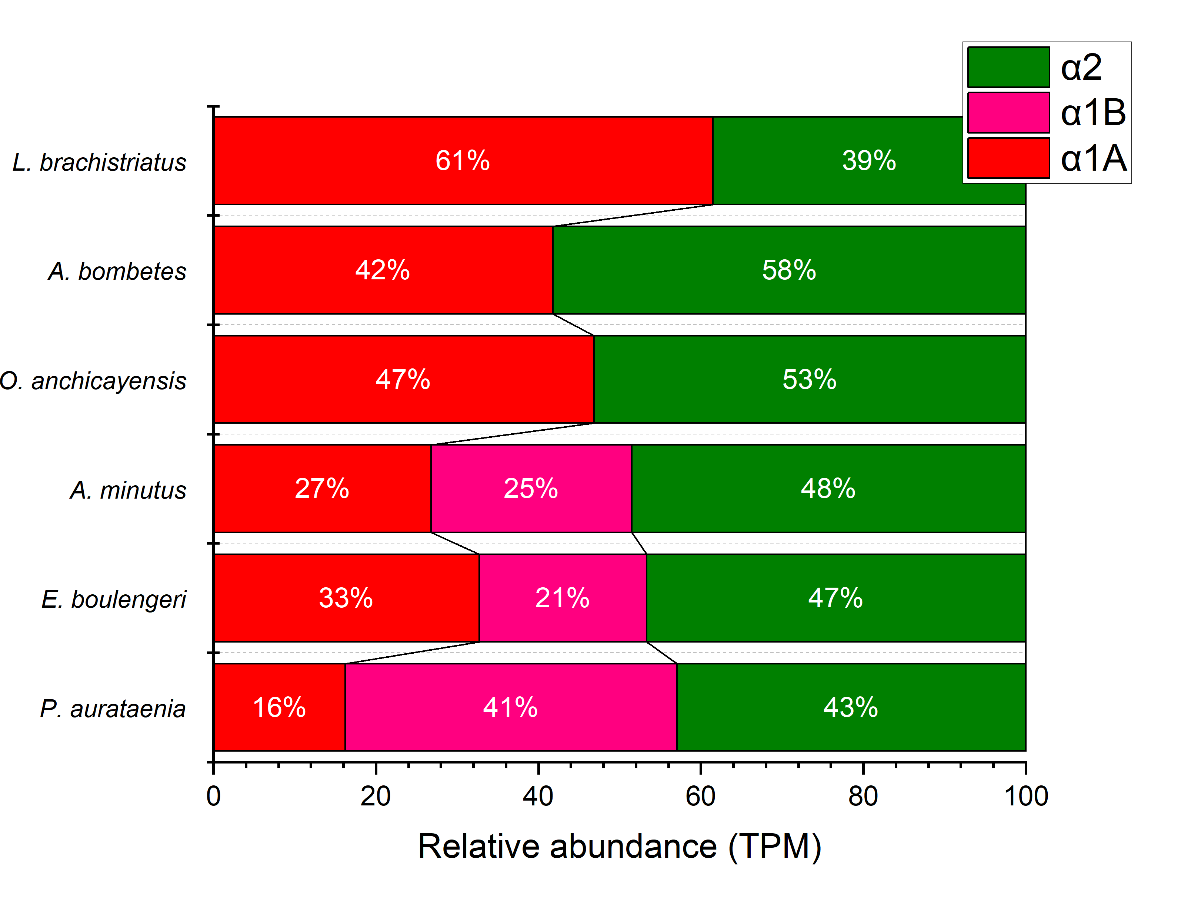


Figure S2. Relative abundances of each transcript encoding α-NKA isoforms in skeletal muscle transcriptomes from dendrobatid species. These proportions were calculated based on the transcripts per million (TPM) values for identified α-NKA isoforms. The figure shows a higher abundance of transcripts encoding isoforms (α_1_A and α_2_) with a susceptible phenotype in *P. aurotaenia*, *E. boulengeri*, and *A. minutus* with respect to α_1_B, whereas *A. bombetes* and *O. anchicayensis* presented a slightly higher abundance of transcript encoding α_2_ isoforms (with 120H substitution) compared to α_1_ (which retains the residues conferring susceptibility to CTS).

Table S1. De novo transcriptome assembly statistics and assembly quality assessment

| Species | No. clean reads | No. Transcripts (Contigs) | Average Contig | Total assembled bases | N50 | Bowtie2 -Overall alignment rate (%) |  |
| --- | --- | --- | --- | --- | --- | --- | --- |
|  |  |  |  |  |  |  |  |
| *P. aurotaenia* | 24775019 | 80121 | 953.72 | 76412880 | 1839 | 98.3 |  |
| *E. boulengeri* | 23842688 | 86972 | 965.42 | 50912919 | 1905 | 98.01 |  |
| *A. minutus* | 20971055 | 93186 | 964.58 | 89885011 | 1912 | 97.69 |  |
| *O. anchicayensis* | 27529856 | 101907 | 933.33 | 95112610 | 1873 | 98.26 |  |
| *A. bombetes* | 27816978 | 105959 | 1010.59 | 107080816 | 2082 | 98.2 |  |
| *L. brachistriatus* | 22559851 | 85462 | 1047.75 | 89542471 | 2104 | 98.21 |  |

Table S2. Descriptive Statistics of the Binding Energies (BE) obtained by Molecular Docking for α-NKA isoform receptors.

| **Receptor*** | **Count** | **Mean** | **SD** | **Median** | **Max** | **Min** |
| --- | --- | --- | --- | --- | --- | --- |
| Ab_ATP1A1 | 30 | -9.42133 | 0.544576 | -9.244 | -8.694 | -10.65 |
| Ab_ATP1A2 | 30 | -8.73683 | 0.423945 | -8.9565 | -7.857 | -9.362 |
| Am_ATP1A1A | 30 | -9.29367 | 0.627009 | -9.3745 | -8.357 | -10.55 |
| Am_ATP1A1B | 30 | -8.55867 | 0.391391 | -8.582 | -7.858 | -9.201 |
| Am_ATP1A2 | 30 | -9.28303 | 0.650935 | -9.3705 | -8.022 | -10.24 |
| ATP1A1_Rhinella | 30 | -8.85007 | 0.667663 | -8.7545 | -7.745 | -9.991 |
| ATP1A2_Rhinella | 30 | -9.8112 | 0.314322 | -9.8035 | -9.24 | -10.526 |
| Cf_ATP1A1 | 30 | -9.12037 | 0.471213 | -9.0515 | -8.408 | -9.97 |
| Cf_ATP1A2 | 30 | -9.1046 | 0.493416 | -9.097 | -8.395 | -9.982 |
| *S. scrofa*_ATP1A1 | 30 | -10.6771 | 0.626962 | -10.7895 | -9.0567 | -11.794 |
| Eb_ATP1A1_A | 30 | -9.4436 | 0.674558 | -9.4825 | -8.519 | -10.69 |
| Eb_ATP1A1_B | 30 | -8.49503 | 0.652117 | -8.443 | -7.554 | -9.804 |
| Eb_ATP1A2 | 30 | -9.31877 | 0.669064 | -9.247 | -8.05 | -10.34 |
| K0G7C4_Dannaus | 30 | -8.5346 | 0.675826 | -8.7115 | -7.394 | -9.585 |
| Oh_ATP1A1 | 30 | -9.33683 | 0.503337 | -9.0995 | -8.767 | -10.85 |
| Oh_ATP1A2 | 30 | -8.7697 | 0.532485 | -8.8015 | -8.063 | -9.779 |
| Pa_ATP1A1A | 30 | -9.40887 | 0.575847 | -9.2885 | -8.582 | -10.55 |
| Pa_ATP1A1B | 30 | -8.94123 | 0.614269 | -8.917 | -8.121 | -9.84 |
| Pa_ATP1A2 | 30 | -9.2249 | 0.585308 | -9.1345 | -8.097 | -10.31 |

* *Andinobates bombetes* (Ab), *Andinobates minutus* (Am), *Colostethus fraterdanieli* (Cf), *Epipedobates boulengeri (Eb), Oophaga histrionica* (Oh), *Phylobates aurotaenia* (Pa); ^a^ Mean of the AutoDock Vina score for 5 replicates.

Table S3. Descriptive Statistics of the BE obtained by Molecular Docking for CTS-NKA complexes.

| **Receptor*** | **Ligand** | **Count** | **Mean** | **SD** | **Median^a^** | **Max** | **Min** |
| --- | --- | --- | --- | --- | --- | --- | --- |
| Ab_ATP1A1 | CMR | 5.0 | -9.9 | 0.1 | -9.9 | -9.6 | -10.0 |
| Ab_ATP1A1 | DIXG | 5.0 | -9.1 | 0.0 | -9.1 | -9.1 | -9.2 |
| Ab_ATP1A1 | OBG | 5.0 | -9.0 | 0.3 | -8.8 | -8.7 | -9.3 |
| Ab_ATP1A1 | OBN | 5.0 | -10.2 | 0.3 | -10.2 | -9.8 | -10.7 |
| Ab_ATP1A1 | OLD | 5.0 | -9.5 | 0.3 | -9.5 | -9.1 | -10.0 |
| Ab_ATP1A1 | SPT | 5.0 | -8.8 | 0.1 | -8.8 | -8.7 | -9.0 |
| Ab_ATP1A2 | CMR | 5.0 | -9.1 | 0.1 | -9.0 | -9.0 | -9.2 |
| Ab_ATP1A2 | DIXG | 5.0 | -8.4 | 0.3 | -8.3 | -8.2 | -9.0 |
| Ab_ATP1A2 | OBG | 5.0 | -8.1 | 0.1 | -8.2 | -8.1 | -8.2 |
| Ab_ATP1A2 | OBN | 5.0 | -8.7 | 0.5 | -8.8 | -7.9 | -9.1 |
| Ab_ATP1A2 | OLD | 5.0 | -9.1 | 0.2 | -9.0 | -9.0 | -9.4 |
| Ab_ATP1A2 | SPT | 5.0 | -9.0 | 0.0 | -9.0 | -8.9 | -9.0 |
| Am_ATP1A1A | CMR | 5.0 | -10.1 | 0.3 | -10.1 | -9.6 | -10.6 |
| Am_ATP1A1A | DIXG | 5.0 | -9.1 | 0.0 | -9.1 | -9.1 | -9.1 |
| Am_ATP1A1A | OBG | 5.0 | -8.6 | 0.1 | -8.6 | -8.6 | -8.7 |
| Am_ATP1A1A | OBN | 5.0 | -9.8 | 0.1 | -9.9 | -9.7 | -9.9 |
| Am_ATP1A1A | OLD | 5.0 | -9.7 | 0.0 | -9.7 | -9.6 | -9.7 |
| Am_ATP1A1A | SPT | 5.0 | -8.5 | 0.1 | -8.5 | -8.4 | -8.6 |
| Am_ATP1A1B | CMR | 5.0 | -8.3 | 0.1 | -8.3 | -8.2 | -8.4 |
| Am_ATP1A1B | DIXG | 5.0 | -7.9 | 0.1 | -8.0 | -7.9 | -8.0 |
| Am_ATP1A1B | OBG | 5.0 | -8.8 | 0.1 | -8.8 | -8.6 | -9.0 |
| Am_ATP1A1B | OBN | 5.0 | -8.9 | 0.2 | -8.9 | -8.6 | -9.0 |
| Am_ATP1A1B | OLD | 5.0 | -8.4 | 0.1 | -8.5 | -8.3 | -8.6 |
| Am_ATP1A1B | SPT | 5.0 | -9.0 | 0.1 | -9.0 | -8.9 | -9.2 |
| Am_ATP1A2 | CMR | 5.0 | -9.8 | 0.3 | -10.0 | -9.4 | -10.2 |
| Am_ATP1A2 | DIXG | 5.0 | -9.0 | 0.0 | -9.0 | -8.9 | -9.0 |
| Am_ATP1A2 | OBG | 5.0 | -8.1 | 0.1 | -8.2 | -8.0 | -8.2 |
| Am_ATP1A2 | OBN | 5.0 | -9.9 | 0.3 | -10.0 | -9.7 | -10.2 |
| Am_ATP1A2 | OLD | 5.0 | -9.6 | 0.2 | -9.6 | -9.3 | -9.9 |
| Am_ATP1A2 | SPT | 5.0 | -9.2 | 0.2 | -9.1 | -9.0 | -9.6 |
| ATP1A1_Rhinella | CMR | 5.0 | -7.9 | 0.1 | -7.9 | -7.7 | -8.0 |
| ATP1A1_Rhinella | DIXG | 5.0 | -8.4 | 0.2 | -8.4 | -8.0 | -8.6 |
| ATP1A1_Rhinella | OBG | 5.0 | -8.9 | 0.2 | -8.8 | -8.7 | -9.3 |
| ATP1A1_Rhinella | OBN | 5.0 | -9.8 | 0.2 | -9.9 | -9.6 | -10.0 |
| ATP1A1_Rhinella | OLD | 5.0 | -9.4 | 0.2 | -9.4 | -9.0 | -9.6 |
| ATP1A1_Rhinella | SPT | 5.0 | -8.8 | 0.2 | -8.8 | -8.7 | -9.2 |
| ATP1A2_Rhinella | CMR | 5.0 | -9.6 | 0.2 | -9.6 | -9.3 | -9.8 |
| ATP1A2_Rhinella | DIXG | 5.0 | -9.8 | 0.1 | -9.8 | -9.7 | -10.0 |
| ATP1A2_Rhinella | OBG | 5.0 | -9.8 | 0.1 | -9.8 | -9.7 | -9.9 |
| ATP1A2_Rhinella | OBN | 5.0 | -10.4 | 0.1 | -10.4 | -10.3 | -10.5 |
| ATP1A2_Rhinella | OLD | 5.0 | -9.8 | 0.1 | -9.9 | -9.7 | -9.9 |
| ATP1A2_Rhinella | SPT | 5.0 | -9.5 | 0.1 | -9.4 | -9.2 | -9.6 |
| Cf_ATP1A1 | CMR | 5.0 | -9.8 | 0.2 | -9.8 | -9.6 | -10.0 |
| Cf_ATP1A1 | DIXG | 5.0 | -8.5 | 0.1 | -8.5 | -8.4 | -8.7 |
| Cf_ATP1A1 | OBG | 5.0 | -8.9 | 0.3 | -8.8 | -8.7 | -9.3 |
| Cf_ATP1A1 | OBN | 5.0 | -9.4 | 0.3 | -9.4 | -9.1 | -10.0 |
| Cf_ATP1A1 | OLD | 5.0 | -9.2 | 0.3 | -9.1 | -8.9 | -9.5 |
| Cf_ATP1A1 | SPT | 5.0 | -8.9 | 0.2 | -8.8 | -8.8 | -9.2 |
| Cf_ATP1A2 | CMR | 5.0 | -9.8 | 0.1 | -9.8 | -9.7 | -10.0 |
| Cf_ATP1A2 | DIXG | 5.0 | -8.7 | 0.0 | -8.7 | -8.7 | -8.7 |
| Cf_ATP1A2 | OBG | 5.0 | -8.5 | 0.1 | -8.4 | -8.4 | -8.5 |
| Cf_ATP1A2 | OBN | 5.0 | -9.5 | 0.3 | -9.4 | -9.0 | -9.9 |
| Cf_ATP1A2 | OLD | 5.0 | -9.0 | 0.2 | -8.9 | -8.8 | -9.2 |
| Cf_ATP1A2 | SPT | 5.0 | -9.1 | 0.0 | -9.2 | -9.1 | -9.2 |
| ATP1A1_*S.scrofa* | CMR | 5.0 | -10.8 | 0.1 | -10.7 | -10.6 | -10.9 |
| ATP1A1_*S.scrofa* | DIXG | 5.0 | -10.9 | 0.1 | -10.9 | -10.7 | -11.0 |
| ATP1A1*_S.scrofa* | OBG | 5.0 | -10.9 | 0.1 | -10.9 | -10.7 | -11.0 |
| ATP1A1*_S.scrofa* | OBN | 5.0 | -11.5 | 0.3 | -11.6 | -11.0 | -11.8 |
| ATP1A1*_S.scrofa* | OLD | 5.0 | -10.5 | 0.1 | -10.5 | -10.4 | -10.6 |
| ATP1A1_*S.scrofa* | SPT | 5.0 | -9.6 | 0.4 | -9.6 | -9.1 | -10.0 |
| Eb_ATP1A1_A | CMR | 5.0 | -10.0 | 0.2 | -10.0 | -9.7 | -10.3 |
| Eb_ATP1A1_A | DIXG | 5.0 | -9.5 | 0.6 | -9.2 | -9.1 | -10.6 |
| Eb_ATP1A1_A | OBG | 5.0 | -8.8 | 0.2 | -8.7 | -8.7 | -9.2 |
| Eb_ATP1A1_A | OBN | 5.0 | -10.2 | 0.4 | -10.3 | -9.6 | -10.7 |
| Eb_ATP1A1_A | OLD | 5.0 | -9.6 | 0.3 | -9.6 | -9.3 | -9.9 |
| Eb_ATP1A1_A | SPT | 5.0 | -8.6 | 0.0 | -8.6 | -8.5 | -8.6 |
| Eb_ATP1A1_B | CMR | 5.0 | -9.1 | 0.6 | -9.3 | -8.4 | -9.8 |
| Eb_ATP1A1_B | DIXG | 5.0 | -7.9 | 0.1 | -7.8 | -7.7 | -8.1 |
| Eb_ATP1A1_B | OBG | 5.0 | -7.7 | 0.2 | -7.7 | -7.6 | -8.0 |
| Eb_ATP1A1_B | OBN | 5.0 | -8.9 | 0.3 | -9.0 | -8.5 | -9.3 |
| Eb_ATP1A1_B | OLD | 5.0 | -9.0 | 0.4 | -9.0 | -8.6 | -9.7 |
| Eb_ATP1A1_B | SPT | 5.0 | -8.3 | 0.2 | -8.2 | -8.1 | -8.7 |
| Eb_ATP1A2 | CMR | 5.0 | -9.7 | 0.3 | -9.8 | -9.4 | -10.0 |
| Eb_ATP1A2 | DIXG | 5.0 | -9.0 | 0.0 | -9.0 | -9.0 | -9.1 |
| Eb_ATP1A2 | OBG | 5.0 | -8.2 | 0.1 | -8.2 | -8.1 | -8.3 |
| Eb_ATP1A2 | OBN | 5.0 | -10.2 | 0.2 | -10.3 | -9.9 | -10.3 |
| Eb_ATP1A2 | OLD | 5.0 | -9.6 | 0.1 | -9.6 | -9.5 | -9.8 |
| Eb_ATP1A2 | SPT | 5.0 | -9.1 | 0.1 | -9.1 | -8.9 | -9.1 |
| K0G7C4_*Dannaus* | CMR | 5.0 | -9.0 | 0.1 | -9.0 | -8.9 | -9.2 |
| K0G7C4_*Dannaus* | DIXG | 5.0 | -7.8 | 0.2 | -7.7 | -7.7 | -8.1 |
| K0G7C4_*Dannaus* | OBG | 5.0 | -7.6 | 0.2 | -7.5 | -7.4 | -7.9 |
| K0G7C4_*Dannaus* | OBN | 5.0 | -9.4 | 0.3 | -9.5 | -9.0 | -9.6 |
| K0G7C4_*Dannaus* | OLD | 5.0 | -8.9 | 0.0 | -8.9 | -8.8 | -8.9 |
| K0G7C4_*Dannaus* | SPT | 5.0 | -8.6 | 0.1 | -8.6 | -8.4 | -8.6 |
| Oh_ATP1A1 | CMR | 5.0 | -10.1 | 0.5 | -10.1 | -9.6 | -10.9 |
| Oh_ATP1A1 | DIXG | 5.0 | -9.1 | 0.0 | -9.1 | -9.0 | -9.1 |
| Oh_ATP1A1 | OBG | 5.0 | -8.8 | 0.1 | -8.8 | -8.8 | -8.9 |
| Oh_ATP1A1 | OBN | 5.0 | -9.6 | 0.2 | -9.7 | -9.3 | -10.0 |
| Oh_ATP1A1 | OLD | 5.0 | -9.3 | 0.3 | -9.3 | -9.0 | -9.8 |
| Oh_ATP1A1 | SPT | 5.0 | -9.1 | 0.0 | -9.1 | -9.0 | -9.1 |
| Oh_ATP1A2 | CMR | 5.0 | -9.5 | 0.2 | -9.5 | -9.2 | -9.8 |
| Oh_ATP1A2 | DIXG | 5.0 | -8.3 | 0.1 | -8.2 | -8.2 | -8.4 |
| Oh_ATP1A2 | OBG | 5.0 | -8.1 | 0.1 | -8.2 | -8.1 | -8.2 |
| Oh_ATP1A2 | OBN | 5.0 | -8.8 | 0.1 | -8.8 | -8.8 | -8.9 |
| Oh_ATP1A2 | OLD | 5.0 | -9.2 | 0.2 | -9.2 | -9.0 | -9.6 |
| Oh_ATP1A2 | SPT | 5.0 | -8.7 | 0.3 | -8.5 | -8.4 | -9.0 |
| Pa_ATP1A1A | CMR | 5.0 | -9.9 | 0.3 | -9.8 | -9.7 | -10.3 |
| Pa_ATP1A1A | DIXG | 5.0 | -9.4 | 0.4 | -9.2 | -9.2 | -10.0 |
| Pa_ATP1A1A | OBG | 5.0 | -8.9 | 0.3 | -8.7 | -8.6 | -9.2 |
| Pa_ATP1A1A | OBN | 5.0 | -10.0 | 0.4 | -10.0 | -9.5 | -10.6 |
| Pa_ATP1A1A | OLD | 5.0 | -9.6 | 0.3 | -9.6 | -9.1 | -10.0 |
| Pa_ATP1A1A | SPT | 5.0 | -8.7 | 0.1 | -8.6 | -8.6 | -8.8 |
| Pa_ATP1A1B | CMR | 5.0 | -9.6 | 0.1 | -9.5 | -9.5 | -9.8 |
| Pa_ATP1A1B | DIXG | 5.0 | -8.2 | 0.0 | -8.2 | -8.1 | -8.2 |
| Pa_ATP1A1B | OBG | 5.0 | -8.7 | 0.2 | -8.9 | -8.4 | -8.9 |
| Pa_ATP1A1B | OBN | 5.0 | -9.3 | 0.5 | -9.3 | -8.6 | -9.8 |
| Pa_ATP1A1B | OLD | 5.0 | -9.5 | 0.1 | -9.5 | -9.3 | -9.6 |
| Pa_ATP1A1B | SPT | 5.0 | -8.3 | 0.1 | -8.3 | -8.2 | -8.5 |
| Pa_ATP1A2 | CMR | 5.0 | -10.0 | 0.4 | -10.3 | -9.4 | -10.3 |
| Pa_ATP1A2 | DIXG | 5.0 | -9.0 | 0.0 | -9.0 | -9.0 | -9.1 |
| Pa_ATP1A2 | OBG | 5.0 | -8.3 | 0.3 | -8.2 | -8.1 | -8.8 |
| Pa_ATP1A2 | OBN | 5.0 | -9.7 | 0.2 | -9.6 | -9.5 | -10.0 |
| Pa_ATP1A2 | OLD | 5.0 | -9.3 | 0.1 | -9.3 | -9.2 | -9.5 |
| Pa_ATP1A2 | SPT | 5.0 | -9.0 | 0.1 | -9.0 | -8.9 | -9.1 |

* *Andinobates bombetes* (Ab), *Andinobates minutus* (Am), *Colostethus fraterdanieli* (Cf), *Epipedobates boulengeri (Eb), Oophaga histrionica* (Oh), *Phylobates aurotaenia* (Pa); ^a^ Mean of the AutoDock Vina score for 5 replicates.

Table S4. Results in a generalized linear model with gamma distribution and Anova (deviation analysis).

|  | Df | Deviance | Resid. Df | Resid. Dev | F | Pr(>F) |
| --- | --- | --- | --- | --- | --- | --- |
| NULL |  |  | 569 | 3.7874 |  |  |
| Receptor | 18 | 1.61661 | 551 | 2.1708 | 151.864 | < 2.2e-16 *** |
| Ligand | 5 | 1.07955 | 546 | 1.0913 | 365.086 | < 2.2e-16 *** |
| Receptor:Ligand | 90 | 0.82255 | 456 | 0.2687 | 15.454 | < 2.2e-16 *** |
| Signif. codes: 0 ‘***’ 0.001 ‘**’ 0.01 ‘*’ 0.05 ‘.’ 0.1 ‘ ’ 1 | | | | | | |

Table S5. Tukey's post-hoc test* for multiple comparisons. The P value indicates the level of significance with α = 0.05

| Comparisson | Estimate | Std. Error | t value | Pr(>\|t\|) * |
| --- | --- | --- | --- | --- |
| Ab_ATP1A1 vs Ab_ATP1A2 | 0.68450 | 0.05711 | 11.985 | < 2e-16 *** |
| Ab_ATP1A1 vs *S.scrofa* ATP1A1 | -1.25579 | 0.06331 | -19.834 | < 2e-16 *** |
| Ab_ATP1A2 vs. *S.scrofa* ATP1A1 | -1.94029 | 0.06133 | -31.637 | < 2e-16 *** |
| Am_ATP1A1A vs Am_ATP1A1B | 0.73500 | 0.05618 | 13.082 | < 2e-16 *** |
| Am_ATP1A1A vs Am_ATP1A2 | 0.01063 | 0.05845 | 0.182 | 1.000000 |
| Am_ATP1A1A vs S.scrofa ATP1A1 | -1.38346 | 0.06296 | -21.974 | < 2e-16 *** |
| Am_ATP1A2 vs S.scrofa ATP1A1 | -1.39409 | 0.06293 | -22.153 | < 2e-16 *** |
| ATP1A1_*Rhinella* vs. S.scrofa ATP1A1 | -1.82706 | 0.06169 | -29.615 | < 2e-16 *** |
| ATP1A2_*Rhinella* vs. S.scrofa ATP1A1 | -0.86592 | 0.06445 | -13.436 | < 2e-16 *** |
| Cf_ATP1A1 vs. Cf_ATP1A2 | 0.01577 | 0.05728 | 0.275 | 1.000000 |
| Cf_ATP1A1 vs ATP1A1_*S. scrofa* | -1.55676 | 0.06243 | -24.936 | < 2e-16 *** |
| Cf_ATP1A2 vs. ATP1A1_*S. scrofa* | -1.57252 | 0.06239 | -25.205 | < 2e-16 *** |
| Eb_ATP1A1_A vs. Eb_ATP1A1_B | 0.94857 | 0.05651 | 16.785 | < 2e-16 *** |
| Eb_ATP1A1_A vs. Eb_ATP1A2 | 0.12483 | 0.05903 | 2.115 | 1.000000 |
| Eb_ATP1A1_A vs. Eb_ATP1A1_B | 0.94857 | 0.05651 | 16.785 | < 2e-16 *** |
| Eb_ATP1A1_A vs. Eb_ATP1A2 | 0.12483 | 0.05903 | 2.11 | 1.0 |
| *S.scrofa* ATP1A1 vs. Eb_ATP1A1_A | 1.23352 | 0.06339 | 19.458 | < 2e-16 *** |
| Oh_ATP1A1 vs. Oh_ATP1A2 | 0.56713 | 0.05695 | 9.959 | < 2e-16 *** |
| S.scrofa ATP1A1 vs. Oh_ATP1A1 | 1.34029 | 0.06306 | 21.254 | < 2e-16 *** |
| S.scrofa vs. Oh_ATP1A2 | 1.90742 | 0.06144 | 31.045 | < 2e-16 *** |
| Pa_ATP1A1A vs. Pa_ATP1A1B | 0.46763 | 0.05773 | 8.101 | 4.25e-13 *** |
| Pa_ATP1A1A vs. Pa_ATP1A2 | 0.18397 | 0.05859 | 3.140 | 0.073846 |
| *S. scrofa* ATP1A1 vs. Pa_ATP1A1A | 1.26826 | 0.06328 | 20.043 | < 2e-16 *** |
| *S. scrofa* ATP1A1 vs. Pa_ATP1A1B | 1.73589 | 0.06194 | 28.026 | < 2e-16 *** |
| *S. scrofa* ATP1A1 vs. Pa_ATP1A2 | 1.45222 | 0.06275 | 23.143 | < 2e-16 *** |

*P-value adjustment: tukey method for comparing a family of 19 estimates
